# *Slc22a23* Proficiency Influences Rat Behavioral Responses After Lysophosphatidylcholine C20:4n6 Administration

**DOI:** 10.3390/pharmaceutics18080975

**Published:** 2026-08-08

**Authors:** Yasuhiro Uchimura, Masakazu Shinohara, Shuhei Kikuchi, Yoshinori Kubo, Shiori Nagaike, Tomoko Kimura, Kosuke Hattori, Tomoji Mashimo, Jun Udagawa

**Affiliations:** 1 Division of Anatomy and Cell Biology, Department of Anatomy, Shiga University of Medical Science, Otsu 520-2192, Shiga, Japands132212@g.shiga-med.ac.jp (S.N.); udagawa@belle.shiga-med.ac.jp (J.U.); 2 Division of Molecular Epidemiology, The Integrated Center for Mass Spectrometry, Graduate School of Medicine, Kobe University, Kobe 650-0017, Hyogo, Japan; mashino@med.kobe-u.ac.jp; 3 Department of Physical Therapy, Faculty of Health Sciences, Kyoto Tachibana University, Kyoto 607-8175, Kyoto, Japan; kimura-to@tachibana-u.ac.jp; 4 Division of Animal Genetics, Laboratory Animal Research Center, Institute of Medical Science, The University of Tokyo, Minato-ku, Tokyo 108-8639, Tokyo, Japan; kohatto@g.ecc.u-tokyo.ac.jp (K.H.); mashimo@ims.u-tokyo.ac.jp (T.M.)

**Keywords:** lysophosphatidylcholine, arachidonic acid, long-chain polyunsaturated fatty acid, solute carrier family, membrane transporter, major facilitator superfamily, animal behavior

## Abstract

**Background:** The solute carrier family 22 member 23 (*Slc22a23*), a gene encoding a membrane-orphan transporter, is irreversibly upregulated in the brains of rats following fetal undernutrition. **Materials and Method:** To identify potential substrates of the SLC22A23 transporter, we analyzed the plasma of both *Slc22a23*-proficient and deficient rats. **Results:** We found that the level of lysophosphatidylcholine a C20:4 (LPC(20:4)) in plasma was more than twofold lower in the *Slc22a23*^−/−^ rats than in the *Slc22a23*^+/+^ rats. We then conducted a series of animal behavioral tests on *Slc22a23*-proficient (*Slc22a23*^+/+^) and deficient (*Slc22a23*^−/−^) rats to investigate the effects of LPC(20:4) administration. We observed considerable effects in both *Slc22a23*^+/+^ and *Slc22a23*^−/−^ rats, with more pronounced effects in the *Slc22a23*^+/+^ rats. These effects included reductions in total distance travelled in an open field test, reductions in access to the novel objects in a novel object recognition test, reductions in sociability to a familiar rat in a social interaction test, and shorter latencies to the target in the Morris water maze test. **Discussion:** These findings suggest that LPC(20:4) administration enhances memory acquisition in both *Slc22a23*^+/+^ and *Slc22a23*^−/−^ rats, though it appears to be more effective in the *Slc22a23*^+/+^ rats. While the SLC22A23 transporter is likely involved in facilitating the transport of LPC(20:4), other transporters may also play a role in its transport in vivo.

## 1. Introduction

Epidemiological studies have demonstrated a significant association between nutritional stress during fetal development and the onset of lifestyle-related non-communicable diseases (NCDs) such as obesity, type 2 diabetes, and psychiatric disorders such as schizophrenia in adulthood [1,2]. Although the developmental origins of health and disease (DOHaD) theory has been proposed based on those epidemiological studies, the molecular mechanisms underpinning the phenotypic changes associated with the DOHaD theory remain largely unknown.

To elucidate the molecular mechanisms underlying behavioral abnormalities induced by prenatal undernutrition [3], we conducted gene expression analyses using embryonic and adult brain tissues and found that the transcript of the solute carrier family 22 member 23 (*Slc22a23*) gene is irreversibly upregulated by undernutritional stress during fetal development [4]. The *Slc22a23* gene belongs to the solute carrier 22 (*Slc22*) family, which comprises more than 30 family member genes [5]. The *Slc22* family genes are conventionally classified into organic anion transporters (OATs) and organic cation transporters (OCTs) based on sequence analysis, with *Slc22a23* classified as one of the OAT-related transporters [5]. While typical SLC22 transporters have 12 α-helical transmembrane domains [6], SLC22A23 lacks N-terminal transmembrane domains, which correspond to transmembrane domains 1 and 2 of the conventional SLC22 transporters. Instead, SLC22A23 has a long N-terminal extracellular region [6]. Consequently, *Slc22a23* is classified into the OAT-related subclade, along with three other genes: *Slc22a17*, *Slc22a18*, and *Slc22a31* [5]. Although *Slc22a18* is classified into the OAT-related subclade, it may not be a true family member, at least based on sequence analysis [5,7]. Additionally, among humans (Homo sapiens), mice (Mus musculus), and rats (Rattus norvegicus), *Slc22a31*—which has the highest homology to *Slc22a17*—appears to be present only in humans, according to a search in the UniProt database (https://www.uniprot.org). Therefore, it is reasonable to conclude that the bona fide members of the OAT-related subclade conserved among mammalian species are *Slc22a17* and *Slc22a23*. Both genes were previously identified as organic cation transporters highly expressed in the brain; *Slc22a17* was named as BOCT1 (brain organic cation transporter 1), and *Slc22a23* was named as BOCT2 [8,9]. Furthermore, before the discovery of BOCT1 and BOCT2, *Slc22a17* was reported to function as a cell-surface receptor for Lipocalin-2 (Neutrophil gelatinase-associated lipocalin), mediating iron uptake into cells [10], through a process of receptor-mediated endocytosis [11]. Because SLC22A23 also possesses an N-terminal extracellular region like SLC22A17, it may function as a cell-surface receptor for receptor-mediated endocytosis. In summary, *Slc22a23* is considered a non-typical member of the *Slc22* family, and its precise function remains unclear. To reveal its precise function in vivo, we generated a *Slc22a23* knockout rat strain [4]. The *Slc22a23* knockout rats exhibited a lean phenotype, increased spontaneous exploratory movements in an open field, and reduced hippocampal volume [4]. These results suggested that *Slc22a23* is involved in metabolism and neurodevelopment. Given that *Slc22a23* is highly expressed in the mammalian brain [8,9], the induction of neurobehavioral phenotypes in *Slc22a23* knockout rats is not surprising; however, its precise function remains unclear.

Identifying the substrate of the SLC22A23 transporter remains a significant challenge. In this study, we hypothesized that *Slc22a23* deficiency leads to impaired uptake of specific metabolites. To investigate this hypothesis, we compared the plasma from *Slc22a23*-proficient and -deficient rats. We identified potential substrates of the SLC22A23 transporter. Subsequently, we assessed whether administering one of the potential substrates influences the animal’s behavior. We then investigated whether the potential substrate could restore some of the cognitive function impaired in *Slc22a23*^−/−^ rats, which exhibit reduced hippocampal volume.

## 2. Materials and Methods

### 2.1. Rat Husbandry

Rats were kept under specific pathogen-free (SPF) condition at room temperature (20–25 °C) under 40–60% relative humidity in the animal facility of Shiga University of Medical Science. They were housed under a 12-h light/dark cycle, with lights switched on at 8:00 a.m. The rats were housed in 27.6 × 44.5 × 20.4 cm polysulfone cages (CLEA Japan, Tokyo, Japan, cat# CL-0128) equipped with a stainless-steel grid lid. The cages contained 1–2 cm of wood granulate bedding (Safe-Lab, Rosenberg, Germany, SAFE^®^ select) along with pulp enrichments (HAMRI, Ibaraki, Japan, CARE FEEAZ). For comparison of plasma between *Slc22a23*-proficient and -deficient rats, plasma samples for mass spectrometry analysis were collected from 16-week-old male rats fed with CE-2 chow (CLEA Japan, Tokyo, Japan; autoclaved) ad libitum (2022 winter-2) [4]. In January 2024, our animal facility switched to MFG chow (Oriental yeast, Tokyo, Japan; 15 kGy gamma ray irradiated). The parental rats (*Slc22a23*^+/−^) used to produce *Slc22a23*^+/+^ and *Slc22a23*^−/−^ siblings for animal behavioral tests (2024 summer) were fed MFG chow ad libitum after weaning. All rats used for the animal behavioral tests were fed MFG chow ad libitum at least one week before conception. In this study, we focused exclusively on male rats for our behavioral experiments because our previous study [4] indicated that male *Slc22a23*^−/−^ rats travelled significantly longer total distances in an open field test compared to their littermate male controls, while no such difference was observed in female rats (see Figures S1C and S3 of the article). The sample size of this study was calculated using the following method: In our previous study [4], at 8 weeks of age, *Slc22a23*^+/+^ rats showed a total distance travelled with an average of 57.4 m and a standard deviation (SD) of 7.7 in the open field test. In contrast, *Slc22a23*^−/−^ rats showed a total distance travelled with an average of 76.7 m and an SD of 17.7 in the same test. To achieve a statistical power of 0.8, a total sample size of 18 rats (9 rats per group) is required. This power analysis is based on an unpaired *t*-test with a significance level (alpha) of 0.05 (two-tailed), conducted using GraphPad Prism 10 (Dotmatics, Boston, MA, USA). While our previous study compared *Slc22a23*^+/+^ rats with *Slc22a23*^−/−^ rats [4], the present study focused on the differences between saline and lysophosphatidylcholine a C20:4n6 (LPC(20:4)) administration within each genotype. Specifically, we examined whether LPC(20:4) exerts an effect by comparing LPC(20:4) injection with saline injection in each genotype. Consequently, it was not possible to precisely estimate the effect size of LPC(20:4) administration before the experiment. The number of animals used in this study is as follows. For the experiment 2024 summer, 11 dams produced 51 male offspring, of which 14 pups were *Slc22a23*^+/+^ and 13 pups were *Slc22a23*^−/−^, both of which were used in the behavioral tests. For experiment 2024 winter, 14 dams produced 78 male offspring, of which 18 pups were *Slc22a23*^+/+^ and 24 pups were *Slc22a23*^−/−^, both of which were used in the behavioral tests. For experiment 2025 spring, 16 dams produced 78 male offspring, of which 20 pups were *Slc22a23*^+/+^ and 19 pups were *Slc22a23*^−/−^, both of which were used in the behavioral tests. For the time-course sampling experiment following intravenous LPC(20:4) administration, 24 *Slc22a23*^+/+^ and 22 *Slc22a23*^−/−^ male rats (8 weeks old) were used. For the preparation of rat primary hippocampal neurons, *Slc22a23*^+/+^ parents (4 dams) yielded 49 *Slc22a23*^+/+^ E17.5 embryos, and *Slc22a23*^−/−^ parents (3 dams) yielded 38 *Slc22a23*^−/−^ E17.5 embryos. The genotyping of these animals was performed as described before [4]. After ear-punching, tail-tips were digested in a buffer (50 mM Tris pH 8, 100 mM EDTA, 100 mM NaCl, 1% SDS) with 0.2 mg/mL proteinase K at 55 °C until fully digested. Genomic DNA was extracted by phenol:chloroform:isoamyl alcohol, precipitated with isopropanol, resuspended in TE, and heated at 95 °C for 10 min to inactivate proteinase K. The PCR genotyping used KOD One Master Mix. Primers (5′-AACAGGTTCTTGTCAACAGG and 5′-GAGGGCCTCATTTCACATTA) flanking exon 4 of *Slc22a23* yield 1045 bp (wild-type) and 622 bp (knockout) bands. The offspring rats were weaned at postnatal day 23. The rats were raised by housing three of them in a single cage. Three days before the animal behavioral test, each rat was housed individually in a single cage. Rats were monitored at least every 3 days. No unexpected adverse events were observed throughout the entire duration of the experiments. Surplus rats were sacrificed by an overdose of carbon dioxide using the euthanasia equipment provided in the animal facility. If any animals were found to be injured during rearing, they were sacrificed by an overdose of carbon dioxide. Before dissection, the rats were anesthetized by 4% isoflurane inhalation (VIATRIS, Tokyo, Japan) and then sacrificed by collecting whole blood from the heart.

### 2.2. Mass Spectrometry of Rat Plasma

*Slc22a23*^+/−^ rats were bred to obtain trios of *Slc22a23*^+/+^, *Slc22a23*^+/−^, and *Slc22a23*^−/−^ offspring (male, *n* = 5 in each group). The littermate trios were housed together in the same cage and fed CE-2 chow (CLEA Japan, Tokyo, Japan). At 16 weeks of age, each rat was housed in a single cage with a wire mesh (to prevent coprophagy). After 16 h of fasting, blood was collected from the heart while the rats were under 4% isoflurane anesthesia. A 500 units/mL heparin solution (Wako, Osaka, Japan) in 0.9% sodium saline (Otsuka Pharmaceutical, Tokyo, Japan) was used to coat the inside of an 18-gauge regular bevel × 1.5-inch needle (Terumo, Tokyo, Japan) and a 5-mL syringe (Terumo, Tokyo, Japan) for blood collection. The plasma was collected and snap frozen after centrifugation at 1500 RCF for 15 min at 4 °C. The sample preparation for mass spectrometry was performed according to the BIOCRATES MxP Quant 500 kit user manual (BIOCRATES, Innsbruck, Austria). In brief, 10 μL of plasma was added directly onto the 96-well plate provided with the kit. After a drying step of 30 min using N_2_ gas, a derivatization step for 1 h using a 5% phenyl isothiocyanate (PITC) solution was performed. After another drying step for 1 h, 300 μL of 5 mM methanolic ammonium acetate was added as extraction solvent, and after shaking the kit plate for 30 min, the contents were filtered into a lower sandwich plate by centrifugation at 200 RCF for 2 min. The sample extracts were diluted for subsequent FIA-MS/MS and UPLC-MS/MS analysis as specified in the kit user manual. Samples were subjected to two UPLC-ESI-MS/MS analyses in multiple reaction monitoring (MRM) mode, followed by two FIA-MS/MS runs. A Shimadzu LC System (Shimadzu, Kyoto, Japan), equipped with a reversed-phase MxP Quant 500 UHPLC Column, was coupled to a Qtrap 6500 Mass Spectrometer (Sciex, Tokyo, Japan). For quantitation, both LC and FIA data were converted and imported directly into the BIOCRATES software, MetIDQ Oxygen (version DB110-3005-290) (BIOCRATES, Innsbruck, Austria), and quantified.

### 2.3. Synthesis of LPC(20:4)

Lysophosphatidylcholine (LPC) has two isomers: 1-acyl-2-lyso PC and 2-acyl-1-lyso PC. Spontaneous and nonenzymatic intra-molecular acyl migration between the sn-1 and sn-2 positions occurs. Sugasini and Subbaiah [12] demonstrated in vitro kinetics showing that starting from 100% of 2-acyl-1-lysoPC molecules, it transformed into 10% of 2-acyl-1-lysoPC a C20:4n6 and 90% of 1-acyl-2-lysoPC a C20:4n6, as well as 22% of 2-acyl-1-lysoPC a C22:6n3 and 78% of 1-acyl-2-lysoPC a C22:6n3, within 24 h at 37 °C in Tris-HCl buffer, pH 7.4. This indicates that the acyl migration from the sn-2 to the sn-1 position is prominent in vitro. In contrast, Sugasini et al. [13] found that 1-lysoPC a C22:6n3 fed animals showed a significant increase in LPC a C22:6n3 in plasma, but unexpectedly, 80% of LPC a C22:6n3 in plasma was 2-acyl-1-lysoPC. Although the underlying mechanism is not understood, the data suggest that acyl migration from the sn-1 to the sn-2 position is prominent in vivo (at least in plasma). Additionally, Sugasini et al. [14] reported that feeding with either 1-acyl-2-lysoPC a C22:6n3 or 2-acyl-1-lysoPC a C22:6n3 both resulted in their accumulation in the brain and improved spatial learning and memory to the same extent. In a separate line of study, Nguyen et al. [15] used 1-acyl-2-lysoPC a C22:6n3 as the substrate to investigate the MFSD2A (SLC59A1) transporter’s role in transporting LPC a C22:6n3 (LPC(22:6)) across the blood–brain barrier. Based on these previous reports, we decided to use 1-acyl-2-lysoPC a C20:4n6 as LPC(20:4) for intravenous administration into rats in this study. The first step to synthesize 1-acyl-2-lyso-phosphatidylcholine a C20:4n6 (1-acyl-2-lysoPC a C20:4n6) was to prepare a solution containing 1.2 mmol (308.7 mg) of glycerophosphocholine (GPC) (BLD Pharmatech, Shanghai, China, cat# BD135811) and 6 mmol (2 mL; specific gravity 0.92) of arachidonic acid (AA) (BLD Pharmatech, Shanghai, China, cat# BD114601) in a 3.5-mL pierce glass vial (Osaka chemical, Osaka, Japan, cat# 5-106-02) with an 8-mm stir bar. The GPC/AA solution was heated to 150 °C for 15 min under N_2_ gas to dissolve GPC. Once the glass vial had reached room temperature, 77.2 mg of Novozym^®^ 435 (MIK Pharm, Tokyo, Japan; an immobilized lipase B from *Candida antarctica*) [16,17], accounting for 25% of the GPC by weight, was added. The glass vial was filled with N_2_ gas and then stirred at 40 °C for 48 h. After the enzymatic reaction, the sample was applied to an open chromatography column (TOP, Kyoto, Japan, cat# 1309-022; 15 × 250 mm), which was filled with Wakogel^®^ C-200 (Wako, Osaka, Japan) in a developing solvent (chloroform/methanol/water at a ratio of 65:25:4 by volume). To identify fractions containing LPC(20:4), 0.5 µL of each fraction was separated on a Silicagel 70 F_254_ TLC plate (Wako, Osaka, Japan) using a developing solvent (chloroform/methanol/water at a ratio of 65:25:4 by volume). To visualize the LPC(20:4) on the plate, the TLC plate was stained with a solution of 5% 12 molybdo (VI) phosphoric acid (Wako, Osaka, Japan) in ethanol. The main fractions containing LPC(20:4) were concentrated using a rotary evaporator. The concentrated sample was applied to a PLC silica gel 60 0.5 mm (Supelco, Darmstadt, Germany) and separated using a developing solvent (chloroform/methanol/water at a ratio of 65:25:4 by volume). To visualize the migration of LPC(20:4) on the PLC plate, one side of the PLC plate (~1 cm) was stained with I_2_. The unstained part of the silica plate containing LPC(20:4) was scraped and collected. Then the LPC(20:4) in the silica was eluted with 100% methanol under sonication. After evaporating the methanol using a rotary evaporator, the LPC(20:4) pellet was dissolved in 100% chloroform (to a concentration of 10 mg/mL) and stored at −30 °C until use. To confirm the molecular weight of the synthesized LPC(20:4), 0.5 µL of the stock solution was analyzed using TOF-MS (Shimadzu AXIMA confidence, Kyoto, Japan) in a matrix of 0.25 mol/L 2,5-dihydroxybenzoic acid (TCI) in methanol. To confirm the chemical structure of the synthesized LPC(20:4), 4 mg of LPC(20:4) was dissolved in 99.8% chloroform-d containing 0.05% tetramethylsilane (Wako, Osaka, Japan). The ^1^H-NMR chemical shift was analyzed using a 400 MHz NMR spectrometer (JEOL, Tokyo, Japan, JNM-ECZ400S). The ^1^H-NMR shift predictions (solvent: CDCl_3_, frequency: 400 MHz) for 1-acyl-2-lysoPC a C20:4n6 and 2-acyl-1-lysoPC a C20:4n6 were conducted using ChemNMR function in ChemDraw software (version 25.0.2) (Revvity Signals Software, Waltham, MA, USA).

### 2.4. Time-Course Sampling Following Intravenous Injection of LPC(20:4)

For the time-course sampling experiment, 24 *Slc22a23*^+/+^ and 22 *Slc22a23*^−/−^ male rats (8 weeks old) were used. Each rat was housed individually in a cage with a wire mesh (to prevent coprophagy) and fasted for 16 h. After 16 h of fasting, baseline fluid and organ samples were collected (t = 0). Then, LPC(20:4) was administered to the remaining rats via the tail-vein at a dose of 5.437 mg (10 µmol)/kg. At 0.5, 2, 6, or 24 h after injection, blood was collected from the heart while the rats were under 4% isoflurane anesthesia. A 500 units/mL heparin solution (Wako, Osaka, Japan) in 0.9% sodium saline (Otsuka Pharmaceutical, Tokyo, Japan) was used to coat the inside of an 18-gauge regular bevel × 1.5-inch needle (Terumo, Tokyo, Japan) and a 5-mL syringe (Terumo, Tokyo, Japan) for blood collection. Plasma was separated by centrifugation at 1500 RCF for 15 min at 4 °C and snap frozen. The hippocampus [18] and the tip of the median lobe of the liver were also collected. All samples were stored at −80 °C until mass spectrometry analysis.

For LPC(20:4) analysis, 10 µL of plasma or ~5 mg of tissue was transferred to a 1.5 mL Eppendorf tube, then 100 μL of MeOH containing 10 µL of the internal standard mixture (SPLASH LIPIDOMIX, 330707, Avanti Polar Lipids, Alabaster, AL, USA) and 500 µL of methyl-tert-butyl ether (MTBE) was added and shaken for one hour at room temperature in the orbital mixer. Then, 200 μL of water was added, and the mixture was vortexed for 20 s. After centrifugation for 10 min at 16,000 RCF, the supernatant was transferred to a new 1.5 mL Eppendorf tube and evaporated to dryness in a centrifugal concentrator. The dried sample was resuspended in 100 μL of a 10% MeOH:10% MeCN:30% water:50% isopropanol solution, then transferred to a glass amber vial with a micro-insert. LC-MS/MS analysis was performed using the optimized MRM conditions with a Qtrap 6500 (Sciex, Tokyo, Japan) equipped with a Shimadzu LC-30AD HPLC system. A Luna 3 µm NH2, 2 × 100 mm column was used to separate the phospholipids. The mobile phase consisted of (A) 2 mM Ammonium Acetate in 7/93 Dichloromethane/Acetonitrile and (B) 2 mM Ammonium Acetate in 50/50 Water/Acetonitrile. A linear gradient was optimized as follows (flow rate, 0.2–0.7 mL/min): 0–2 min, 0% B; 2–12.5 min, 0–100% B; 12.5–15.0 min, 100% B; 15.0–17.0 min, 0% B. A typical run time was 17 min.

For total fatty acid analysis, nonadecanoic acid (C19:0) was added as an internal standard to each plasma sample (50 µL) or ~5 mg of tissue, followed by total fatty acid extraction, methylester derivatization, and purification using the fatty acid methylation/purification kit (Nacalai Tesque, Kyoto, Japan) according to the manufacturer’s instructions. The methylester-derivatized fatty acid after purification was reconstituted with 100 µL of hexane for subsequent analysis. Fatty acids were analyzed using a gas chromatography/mass spectrometry (QP2010 Ultra, Shimadzu, Kyoto, Japan). The capillary column used for fatty acid separation was SP-2650 (100 m length × 0.25 mm inner diameter × 0.20 µm film thickness, Sigma-Aldrich, St Louis, MO, USA). The column oven temperature was elevated from 140 °C to 240 °C, and the separated fatty acid methylester was detected using a mass spectrometry. The standard mixture of methylester fatty acids was obtained from Sigma-Aldrich.

### 2.5. Behavioral Tests

#### 2.5.1. Open Field (OF) Test

**Figure 1:**
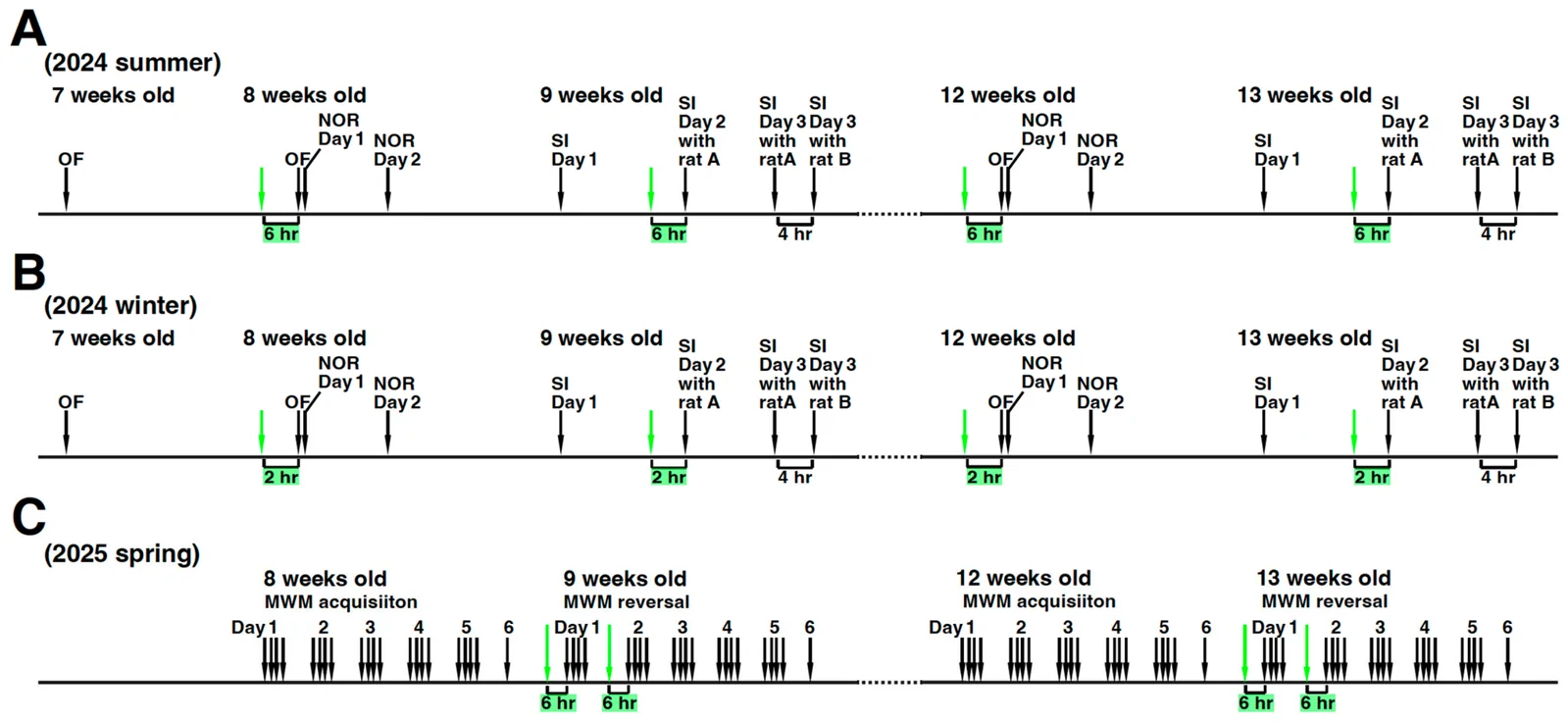
Experimental schedule: (**A**) 2024 summer. Naïve rats were tested in the open field (OF) at 7 weeks of age. A dose of 5.437 mg (10 µmol)/kg of lysophosphatidylcholine a C20:4 (LPC(20:4)) or 0.9% sodium saline (control) was administered via intravenous tail injection 6 h before the OF test (at 8 and 12 weeks of age; green arrows) and on day 2 of the social interaction (SI) test (at 9 and 13 weeks of age; green arrows). Day 1 of the novel object recognition (NOR) test was conducted immediately after the OF test. The number of animals used in each group was as follows: *Slc22a23*^+/+^ control (*n* = 7), *Slc22a23*^+/+^ with LPC(20:4) injection (*n* = 7), *Slc22a23*^−/−^ control (*n* = 7), and *Slc22a23*^−/−^ with LPC(20:4) injection (*n* = 6). The total number of animals used in this experiment was 27. (**B**) 2024 winter. The same schedule as in (**A**), but LPC(20:4) or 0.9% sodium saline was administered 2 h before the tests (green arrows). The number of animals used in each group was as follows: *Slc22a23*^+/+^ control (*n* = 9), *Slc22a23*^+/+^ with LPC(20:4) injection (*n* = 9), *Slc22a23*^−/−^ control (*n* = 12), and *Slc22a23*^−/−^ with LPC(20:4) injection (*n* = 12). The total number of animals used in this experiment was 42. (**C**) 2025 spring. At 9 and 13 weeks of age, LPC(20:4) or 0.9% sodium saline was injected 6 h before the reversal phase on days 1 and 2 in the Morris water maze (MWM) test. The number of animals used in each group was as follows: *Slc22a23*^+/+^ control (*n* = 10), *Slc22a23*^+/+^ with LPC(20:4) injection (*n* = 10), *Slc22a23*^−/−^ control (*n* = 10), and *Slc22a23*^−/−^ with LPC(20:4) injection (*n* = 9). The total number of animals used in this experiment was 39.

**Figure 2:**
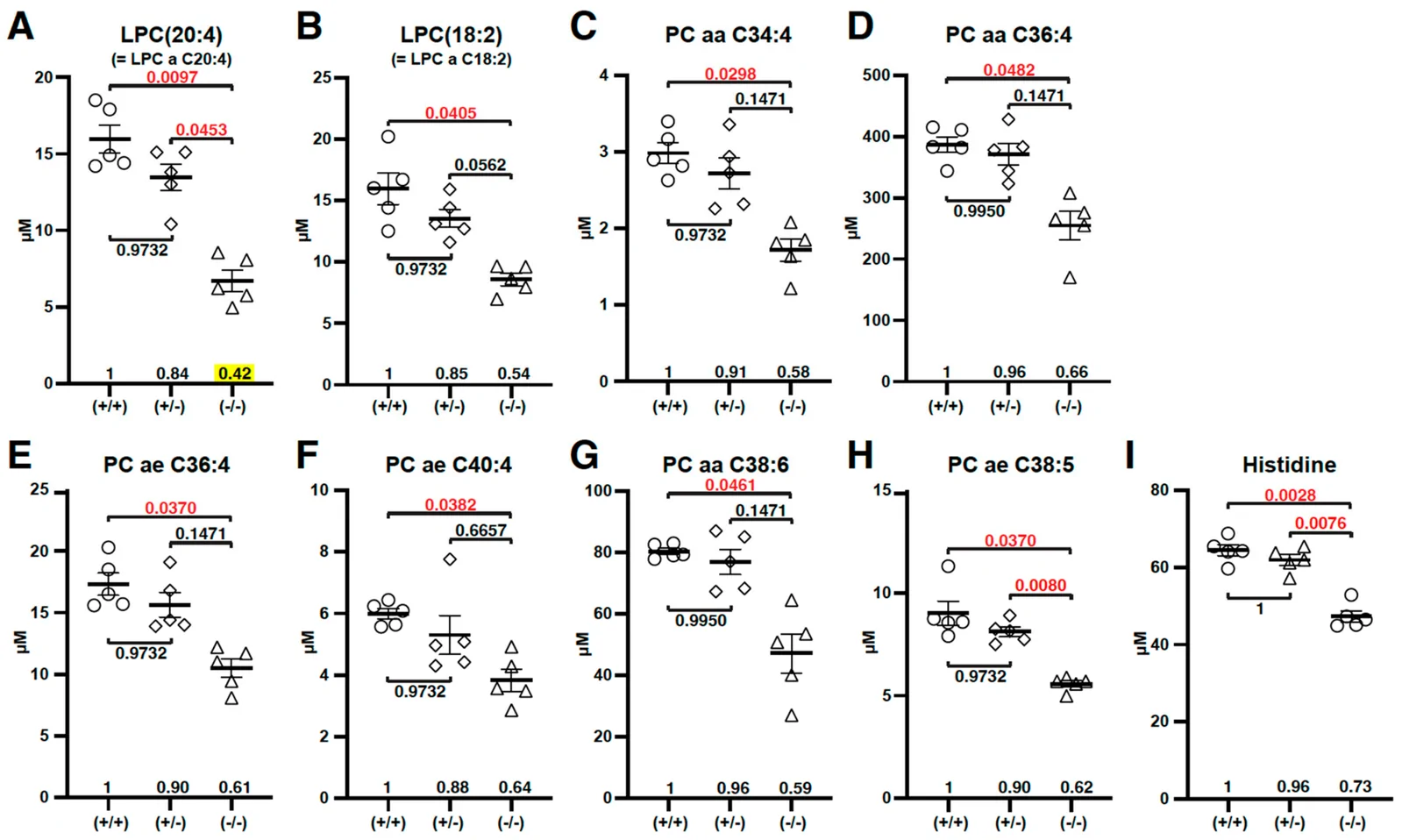
Significantly altered molecules in plasma between *Slc22a23*-proficient and -deficient rats: (**A**–**H**) Lipophilic molecules. Significantly altered lipophilic molecules in the plasma of *Slc22a23*^+/+^, *Slc22a23*^+/−^ and *Slc22a23*^−/−^ rats (*n* = 5 in each group) were identified through mass spectrometry analysis. The total number of rats used in the experiment was 15. The mass spectrometry analysis detected 524 lipophilic molecules (Table S1). Concentrations were estimated based on counted signals, with cinnamic acid as the internal standard. The q-values shown in the graph were adjusted using the Benjamini-Hochberg (BH) method to correct for multiple comparisons of the analyzed metabolites. The error bars represent the mean ± SEM. LPC a C20:4 (=LPC(20:4)) and LPC a C18:2 (=LPC(18:2)) are monoacyl (a) glycerophosphocholines (GPCs) (**A**,**B**). PC aa C34:4, PC aa C36:4, and PC aa C38:6 are diacyl (aa) GPCs (**C**,**D**,**G**). PC ae C36:4, PC ae C40:4, and PC ae C38:5 are esterified and etherified forms of diacyl (ae) GPCs (**E**,**F**,**H**). (**I**) Hydrophilic molecule. Significantly altered hydrophilic molecules in the plasma of *Slc22a23*^+/+^, *Slc22a23*^+/−^ and *Slc22a23*^−/−^ rats (*n* = 5 in each group) were identified through mass spectrometry analysis. The total number of rats used in the experiment was 15. The mass spectrometry analysis detected 84 hydrophilic molecules (Table S2). Concentrations were estimated based on counted signals, with cinnamic acid as the internal standard. The q-values shown in the graph were adjusted using the Benjamini–Hochberg (BH) method to correct for multiple comparisons of the analyzed metabolites. The error bars represent the mean ± SEM. The descriptive statistics are shown in Table S3.

The OF test was conducted in a gray polyvinyl chloride circular chamber (100 cm diameter, 45 cm height). The brightness of the surface of the OF was set to less than 8 lux using a digital lux meter (ONGi Japan, Tokyo, Japan, LUX-275). At 7 weeks of age, the rats of each genotype were divided into two groups (naïve (C) for control, naïve (L) for LPC(20:4) injection) based on their body weight ranking to ensure counterbalancing within each genotype. Here, “naïve” refers to rats that have not yet received any injections, either LPC(20:4) or sodium saline. To minimize potential confounders such as the order of treatments and measurements, the order of animals analyzed was determined based on the order of ear punch procedures before their genotype and body weight ranking were established. Each naïve test rat was placed individually in the center of the OF. The movement of a test rat (movements of more than 1 cm) was recorded using a CompACT video tracking system (Muromachi Kikai, Tokyo, Japan) for 10 min. After each rat was tested, the OF was sprayed with a 10% *v*/*v* ethanol solution and wiped with disposable paper towels. At 8 and 12 weeks of age, a dose of 5.437 mg (10 µmol)/kg of LPC(20:4) or 0.9% sodium saline (Otsuka Pharmaceutical, Tokyo, Japan) was injected via tail-vein 6 h before the OF test (Figure 1A) or 2 h before the OF test (Figure 1B). Since the plasma concentration of LPC(20:4) in *Slc22a23*^−/−^ rats was approximately 10 µM lower than that of *Slc22a23*^+/+^ rats, a dose of 10 µmol/kg of LPC(20:4) was administered to elevate LPC(20:4) levels by approximately 10 µM in the whole body. For the intravenous injection, chloroform from the 10 mg/mL LPC(20:4) stock solution was evaporated completely for 1 h using a rotary evaporator. Then the resulting pellet was dissolved in 0.9% sodium saline (Otsuka Pharmaceutical, Tokyo, Japan) to prepare a 5.437 mg/mL LPC(20:4) solution. A dose of 5.437 mg (10 µmol)/kg of LPC(20:4) was injected into each rat via the tail-vein using a 24-gauge regular bevel × 1-inch needle (Terumo, Tokyo, Japan) and a 1-mL syringe (Terumo, Tokyo, Japan) while the rat was placed in a restraint plastic case.

#### 2.5.2. Novel Object Recognition (NOR) Test

At 8 and 12 weeks of age, immediately after the OF test (Figure 1A,B), acclimation toward Object X as a familiar object was conducted in the OF (100 cm diameter, 45 cm height) for 10 min. In the previous study [4], we could not obtain data from some of the test rats because they climbed onto the lids of the glass bottles. To prevent the test rats from climbing onto the lids of the glass bottles, we attached caps to the bottles. Three types of objects were used in the NOR test. Object X was a shaded round glass bottle (Corning, NY, USA) and capped with a HI-TS PVC 50 × 25 mm pipe. Object Y (a novel object at 8 weeks of age) was a transparent square glass bottle (Iwaki, Shizuoka, Japan) filled with 3A 1/8 molecular sieves (Wako, Osaka, Japan) and capped with a PVC T-shaped 50 mm pipe. Object Z (a novel object at 12 weeks of age) was a transparent round glass bottle (Corning, NY, USA) filled with clear marbles (Matsuno hobby, Osaka, Japan; 17 mm diameter) and capped with a PVC T-shaped 50 mm pipe. On day 1, two identical objects (object X) were placed in the OF. The centers of each object were 25 cm from the wall and 50 cm from each other. Then, a test rat was acclimatized to the field for 10 min. On day 2, two objects were placed in the OF. One object was the same as that used on day 1 (object X) as a familiar object. The other object was object Y, which was used at 8 weeks of age, or object Z, which was used at 12 weeks of age. A test rat was initially placed in the center of the field. The movement of a test rat (movements of more than 1 cm) was recorded using a CompACT video tracking system (Muromachi Kikai, Tokyo, Japan) for 10 min. The duration of access to each object was defined as the time when the head of a test rat was within 7 cm of the wall of each object. The discrimination (d2) ratio was calculated using the following formula: d2 = [time(novel object) − time(familiar object)]/time(novel object + familiar object) [19]. After each rat was tested, the OF and the objects were sprayed with a 10% *v*/*v* ethanol solution and wiped with disposable paper towels.

#### 2.5.3. Social Interaction (SI) Test

At 9 and 13 weeks of age, a SI test was conducted using two intruder rats in a gray polyvinyl chloride circular chamber (150 cm diameter, 45 cm height). The brightness of the field surface was set to less than 8 lux using a digital lux meter (ONGi Japan, Tokyo, Japan, LUX-275). Two identical circular cages (20 cm diameter, 20 cm height) made of stainless-steel wire were used to house a male intruder rat. The two intruder rats (rat A and rat B) were male Slc:Wistar rats purchased from an animal supplier (Japan SLC, Shizuoka, Japan). The intruder rats were kept isolated from the test rats until the SI test was performed. On day 1, two empty circular cages were placed in the field. The centers of each cage were 30 cm from the wall and 90 cm from each other. On day 2, a dose of 5.437 mg (10 µmol)/kg of LPC(20:4) or 0.9% sodium saline (Otsuka Pharmaceutical, Tokyo, Japan) was injected via tail-vein 6 h before the SI test (Figure 1A) or 2 h before the SI test (Figure 1B). At the time of the SI test on day 2, an intruder rat (rat A) was placed in one of the stainless-steel cages, and then a test rat was placed in the center of the field. The movement of a test rat (movements of more than 1 cm) was recorded using a CompACT video tracking system (Muromachi Kikai, Tokyo, Japan) for 10 min. The first test on day 3 was carried out as on day 2, but without any injection. For the second test on day 3, the intruder rat was replaced by rat B. The approach to a target object was measured as the duration of access when the head of a test rat was within 7 cm of the wall of the object. The discrimination ratio (d2) was calculated using the following formula: d2 = [time(quest cage) − time(empty cage)]/time(quest cage + empty cage). After each rat was tested, the field and the stainless-steel cages were sprayed with 10% *v*/*v* ethanol solution and wiped with disposable paper towels.

#### 2.5.4. Morris Water Maze (MWM) Test

A MWM test was conducted following the protocol established by Vorhees and Williams [20], with some modifications (Figure 1C, Table 1). Our MWM test was conducted in a gray polyvinyl chloride circular chamber (150 cm diameter, 45 cm height). A black platform (8 cm diameter, 18 cm height) was set up with its center positioned 30 cm from the wall. The black platform was placed in the west (W), east (E), south (S), and north (N) positions during the trials from day 1 to day 5 at 8, 9, 12, and 13 weeks of age, respectively. The circular chamber was filled with tap water stained with a black watercolor paint (Pentel, Tokyo, Japan, cat# YNG3T28), with the water surface 3 cm above the black platform. The water temperature was maintained at 20 ± 1 °C. An electric bulb was positioned on the floor, facing upward toward the ceiling. The brightness of the water surface was set to 3 lux using a digital lux meter (ONGi Japan, Tokyo, Japan, LUX-275). The MWM test was conducted in a room containing various distal cues. At 8 weeks of age, the rats of each genotype were divided into two groups (naïve (C) for control, naïve (L) for LPC(20:4) injection) based on their body weight ranking. To minimize potential confounders such as the order of treatments and measurements, the order of animals analyzed was determined based on the order of ear punch procedures before their genotype and body weight ranking were established. Then, the rats were trained for 5-consecutive-days of the acquisition phase (four trials in each day; each trial lasted up to 120 s, with 15 s interval between trials), and a probe session (30 s without a platform) was conducted on the 6th day. Subsequently, at 9 weeks of age, the rats were tested for their ability to learn a new position in the reversal phase. On days 1 and 2 of the reversal phase, a dose of 5.437 mg (10 µmol)/kg of LPC(20:4) or 0.9% sodium saline (Otsuka Pharmaceutical, Tokyo, Japan) was injected via the tail-vein 6 h before the MWM test. At 12 and 13 weeks of age, the same MWM test was conducted, with the position of the platform in the circular chamber rotated 90°. The movement of a test rat (movements of more than 1 cm) was recorded using a CompACT video tracking system (Muromachi Kikai, Tokyo, Japan). The latency to target (in seconds) was determined when a test rat stayed on the platform for more than 2 s, with the recorded time reduced by those 2 s.

**Table 1:** Morris Water Maze spatial (hidden platform) starting positions used in this research.

	Day (Trial Limit)	Trial 1	Trial 2	Trial 3	Trial 4
Acquisition * (8 weeks)	1 (up to 2 min)	NE	SE	S	N
	2 (up to 2 min)	S	NE	N	SE
	3 (up to 2 min)	N	S	SE	NE
	4 (up to 2 min)	SE	N	NE	S
	5 (up to 2 min)	NE	S	SE	N
	6 (up to 30 s) †	E			
Reversal (9 weeks)	1 (up to 2 min)	SW	NW	N	S
	2 (up to 2 min)	N	SW	S	NW
	3 (up to 2 min)	S	N	NW	SW
	4 (up to 2 min)	NW	S	SW	N
	5 (up to 2 min)	SW	N	NW	S
	6 (up to 30 s) †	W			
Acquisition ‡ (12 weeks)	1 (up to 2 min)	NW	NE	E	W
	2 (up to 2 min)	E	NW	W	NE
	3 (up to 2 min)	W	E	NE	NW
	4 (up to 2 min)	NE	W	NW	E
	5 (up to 2 min)	NW	E	NE	W
	6 (up to 30 s) †	N			
Reversal (13 weeks)	1 (up to 2 min)	SE	SW	W	E
	2 (up to 2 min)	W	SE	E	SW
	3 (up to 2 min)	E	W	SW	SE
	4 (up to 2 min)	SW	E	SE	W
	5 (up to 2 min)	SE	W	SW	E
	6 (up to 30 s) †	S			

N = north, NE = northeast, E = east, SE = southeast, S = south, SW = southwest, W = west, and NW = northwest. The green color indicates the days on which LPC(20:4) was administered 6 h before the MWM tests. * The direction was simply rotated 45° clockwise from the original protocol [20]. † On day 6, the probe trials were performed without the platform. ‡ The direction was rotated 90° clockwise from the reversal phase at 9 weeks of age.

### 2.6. LPC(20:4) Incorporation Assay Using Rat Primary Hippocampal Neurons

To obtain E17.5 embryos, breeding pairs were set up (female *Slc22a23*^+/+^ crossed with male *Slc22a23*^+/+^, and female *Slc22a23*^−/−^ crossed with male *Slc22a23*^−/−^). Vaginal plugs were checked each morning after mating. On gestational day 17.5, pregnant dams were anesthetized with 4% isoflurane, and the uterus was isolated. Embryos were collected from the amniotic sac. Four *Slc22a23*^+/+^ dams produced 14, 12, 12, 11 embryos, and three *Slc22a23*^−/−^ dams produced 13, 13, 12 embryos, respectively. The position of the hippocampus isolated from the embryonic brain was determined according to the “dissociating the hippocampus” method described by Yang et al. [18]. Subsequent steps followed Sahu et al. [21], with modifications to accommodate reagents available in Japan. Hippocampi were collected in a preparation medium consisting of HBBS(−) (Nacalai Tesque, Kyoto, Japan), 1 mM sodium pyruvate (Nacalai Tesque, Kyoto, Japan), and 10 mM HEPES (Nacalai Tesque, Kyoto, Japan). The isolated hippocampi were transferred to a papain solution (0.1 mg/mL papain [Sigma, cat# P4762], 2 µg/mL DNase I [Sigma, cat# D4527] in a stock papain buffer (0.2 mg/mL DL-Cysteine HCl [Wako], 0.2 mg/mL BSA [Sigma], 5 mg/mL Glucose [Nacalai] in D-PBS(-) [Nacalai]), and digested for 10 min at 37 °C. After 3 rounds of trituration in 3 mL of a trituration medium (1 µg/mL DNase I in the preparation medium described above), hippocampal cells were collected by centrifugation at 156 RCF for 5 min. Cells were plated at a density of 0.75 × 10^6^ cells per 35-mm dish (NEST Biotechnology, Wuxi, China, cat# 706001) in 2 mL of a growing medium (Neurobasal^TM^ plus medium [Gibco, Waltham, MA, USA], 2% B27 supplement [Gibco], 1% GlutaMAX-I [Gibco], 1% penicillin-streptomycin [Wako]) on dishes pre-coated with poly-L-lysine (Nacalai Tesque, Kyoto, Japan, cat# 28356-84). After culturing for 7 days, the medium was removed, and the cell surface was washed once with D-PBS(+) (Nacalai Tesque, Kyoto, Japan). Then, 2 mL of D-PBS(+) containing 0, 10, or 100 µM of LPC(20:4) was added. After 5, 30, or 60 min of incubation at 37 °C, the cell surface was washed twice with D-PBS(+). Then, the cells were collected by scraping after the addition of 100% methanol and stored at −80 °C until mass spectrometry analysis. The 50% of the scraped sample was employed for LPC analysis, and the remaining 50% was employed for total fatty acid analysis (described above). Precipitated protein was resuspended in Laemmli buffer (Sigma-Aldrich) and quantified using BCA Protein Assay Kits (Thermo Scientific, Waltham, MA, USA) to adjust for protein concentration.

### 2.7. Statistical Analysis

For the analysis of the comprehensive mass spectrometry dataset, two-tailed *t*-tests were performed in Excel (Microsoft, Redmond, WA, USA). For the correction of multiple comparisons of the mass spectrometry analysis, the *p*-values were adjusted by the Benjamini–Hochberg (BH) method using the “p.adjust” function in R (RStudio version 2025.09.2+418, R version 4.5.2 (2025-10-31)). Other statistical analyses, such as Tukey HSDs, one-tailed *t*-tests, two-tailed *t*-tests, paired two-tailed *t*-tests, and Wilcoxon/Kruskal–Wallis tests, were conducted using JMP pro 18 (JMP Statistical Discovery, Cary, NC, USA). Descriptive statistics, including mean, median, standard deviation (SD), skewness, and 95% confidence interval (CI), were calculated using GraphPad Prism 10 (Dotmatics, Boston, MA, USA). All figures were generated using GraphPad Prism 10 (Dotmatics, Boston, MA, USA). No criteria were set for including and excluding animals (or experimental units) during the experiment. For each experimental group, there were no exclusions of animals, experimental units, or data points in the analysis unless otherwise specified in the figure legends. The behavioral comparisons are unadjusted for multiplicity. The protocols, including the research questions, key design features, and analysis plan, were prepared before the conduct of this study (Figure 1). Since the investigator (Y.U.) was aware of the group allocations, the conduct of the experiments, the outcome assessments, and the data analyses, this study was not conducted in a blinded manner.

## 3. Results

### 3.1. Comparison of Plasma Between Slc22a23-Proficient and -Deficient Rats

To identify the substrate of the SLC22A23 transporter, we analyzed metabolites in the plasma from *Slc22a23*-proficient (*Slc22a23*^+/+^ and *Slc22a23*^+/−^) and *Slc22a23*-deficient (*Slc22a23*^−/−^) rats using mass spectrometry. Through comprehensive mass spectrometry analysis of 524 lipophilic compounds (Table S1) and 84 hydrophilic compounds (Table S2), we discovered that 9 molecules were statistically significantly reduced in the plasma of *Slc22a23*^−/−^ rats compared to *Slc22a23*^+/+^ rats (Figure 2). Among 9 molecules, only 3 molecules—lysophosphatidylcholine a C20:4 (LPC(20:4)) (Figure 2A), phosphatidylcholine ae C38:5 (PC ae C38:5) (Figure 2H) and histidine (Figure 2I)—were consistently and significantly reduced in both comparisons (*Slc22a23*^+/+^ vs. *Slc22a23*^−/−^, *Slc22a23*^+/−^ vs. *Slc22a23*^−/−^). Notably, LPC(20:4) was the only molecule that exhibited more than twofold lower concentration compared to WT, with a ratio of *Slc22a23*^−/−^/*Slc22a23*^+/+^ = 0.42 (Figure 2A; highlighted yellow). Given that LPC(20:4) was distinctly affected by the *Slc22a23* knockout, we decided to investigate whether LPC(20:4) is a substrate of the SLC22A23 transporter.

The molecular weight of LPC(20:4) (*m*/*z* of 544) indicates that the lysolipid consists of a single chain of arachidonic acid (AA) ester bound to a glycerophosphocholine (GPC). The identified LPC(20:4) could be 1-acyl-2-lyso-phosphatidylcholine C20:4n6 (1-acyl-2-lysoPC a C20:4n6), 2-acyl-1-lyso-phosphatidylcholine C20:4n6 (2-acyl-1-lysoPC a C20:4n6), or a mixture of both. LPC(20:4) can produce AA by hydrolysis of the ester bond. The resultant AA is a crucial long-chain polyunsaturated fatty acid (LC-PUFA) in the mammalian brain. AA, or its precursor linoleic acid (C18:2n6), must be obtained from dietary sources [22]. The majority of AA synthesis from linoleic acid takes place in the liver [23], after which AA must be transported to the brain. Since the LPC(20:4) is a water-soluble phospholipid, it is an ideal carrier for transporting AA in the plasma. However, the transport of LPC(20:4) across the plasma membrane, particularly at the blood–brain barrier, requires a specific transporter that is expressed on the plasma membrane. The SLC22A23 transporter may serve as one such transporter for LPC(20:4). To explore this possibility, we investigated whether the *Slc22a23*-deficiency influences the effects of LPC(20:4) administration in vivo.

### 3.2. The NMR Spectrum and Molecular Mass of the Synthesized LPC(20:4)

**Figure 3:**
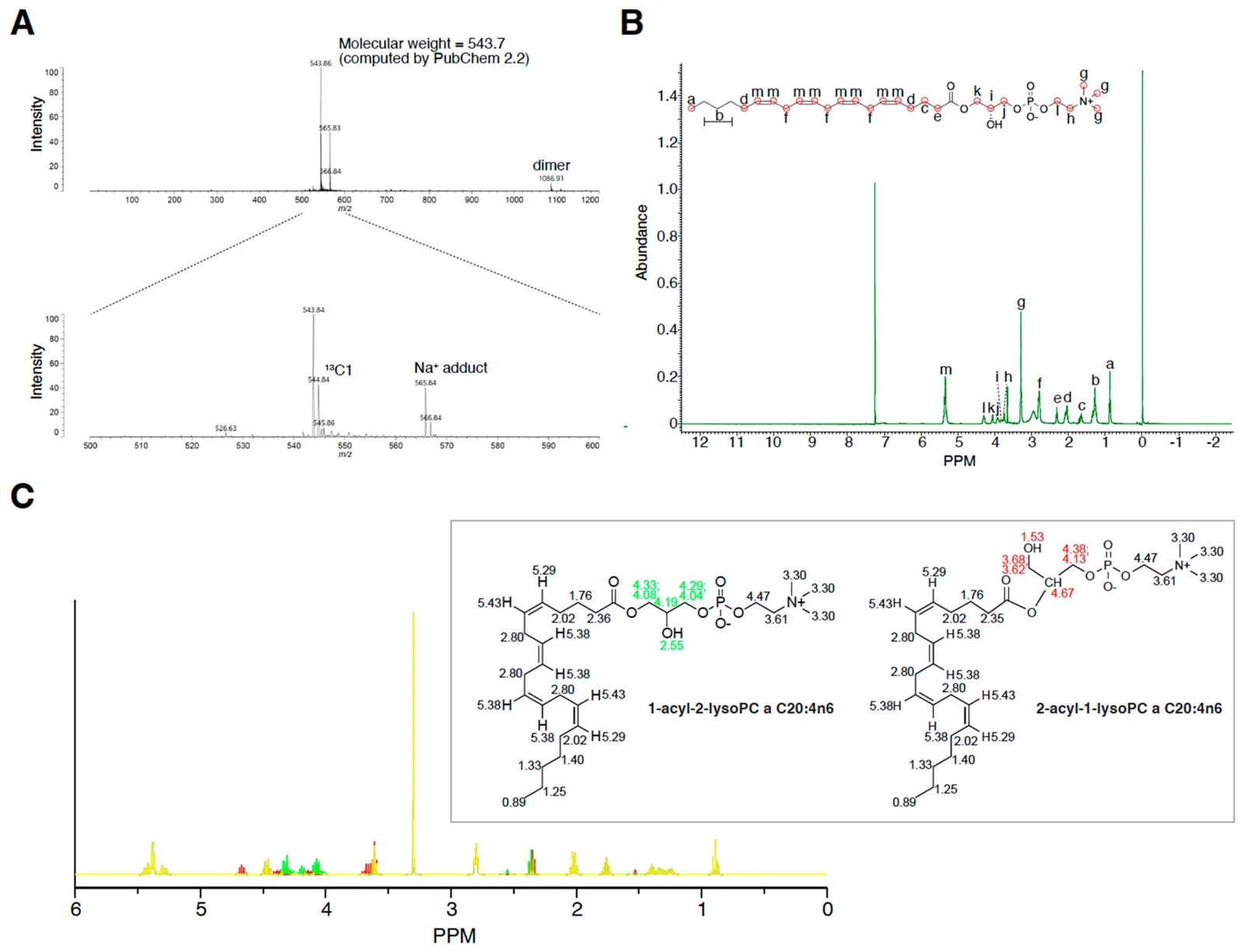
Synthesis of LPC(20:4): (**A**) Time-of-Flight Mass Spectrometry (TOF-MS) of LPC(20:4). Synthesized LPC(20:4) (5 µg) in 0.25 mol/L 2,5-dihydroxybenzoic acid was ionized. The time of flight was measured using reflectron mode (a positive ion mode) (Shimadzu AXIMA confidence). (**B**) ^1^H-NMR of LPC(20:4). Synthesized LPC(20:4) (4 mg) was dissolved in 99.8% chloroform-d containing 0.05% tetramethylsilane. The ^1^H-NMR chemical shift was analyzed using a 400 MHz NMR spectrometer (JEOL, JNM-ECZ400S). The ^1^H-NMR peaks (from “a” to “m”) in the graph were annotated to the positions of proton atoms in the chemical structure of LPC(20:4) showed in the top inset. (**C**) An overlay of the signal patterns of the ^1^H-NMR shift for 1-acyl-2-lysoPC a C20:4n6 (depicted in green) and 2-acyl-1-lysoPC a C20:4n6 (depicted in red) predicted by ChemNMR in ChemDraw software (version 25.0.2). The overlap of the two signals (green and red) appeared as yellow. The inset, at the top right, shows the predicted chemical shift values for the proton atoms at each position. The protons on the glycerol backbone, which provide the major differences between 1-acyl-2-lysoPC a C20:4n6 (depicted in green) and 2-acyl-1-lysoPC a C20:4n6 (depicted in red) are highlighted.

The synthesized LPC(20:4), which refers to LPC(C20:4n6) in this study, was verified through mass spectrometry and ^1^H-NMR spectrometry. The molecular weight was confirmed using Time-of-Flight Mass Spectrometry (TOF-MS). The major peak was within 1 *m*/*z* of the predicted mass of 543.7 *m/z* (Figure 3A). The chemical structure was verified by the chemical shift of the protons in the ^1^H-NMR spectrum (Figure 3B). To assign the peaks in the ^1^H-NMR spectrum, ^1^H-NMR spectrum of LPC [24] and a predicted ^1^H-NMR spectrum of AA (HMDB0001043; https://hmdb.ca/spectra/nmr_one_d/333737#spectrum (accessed on 19 October 2023)) were used as references. The ^1^H-NMR shift predictions (solvent: CDCl_3_, frequency: 400 MHz) for 1-acyl-2-lysoPC a C20:4n6 and 2-acyl-1-lysoPC a C20:4n6 using ChemNMR function in ChemDraw software indicate that the majority of the synthesized LPC(20:4) is 1-acyl-2-lysoPC a C20:4n6 (Figure 3C). In the preparation process, 830 mg of LPC(20:4) was obtained from 5.2 g of GPC, corresponding to a yield of 16%.

### 3.3. Spatial and Time-Course Metabolomics After LPC(20:4) Administration

**Figure 4:**
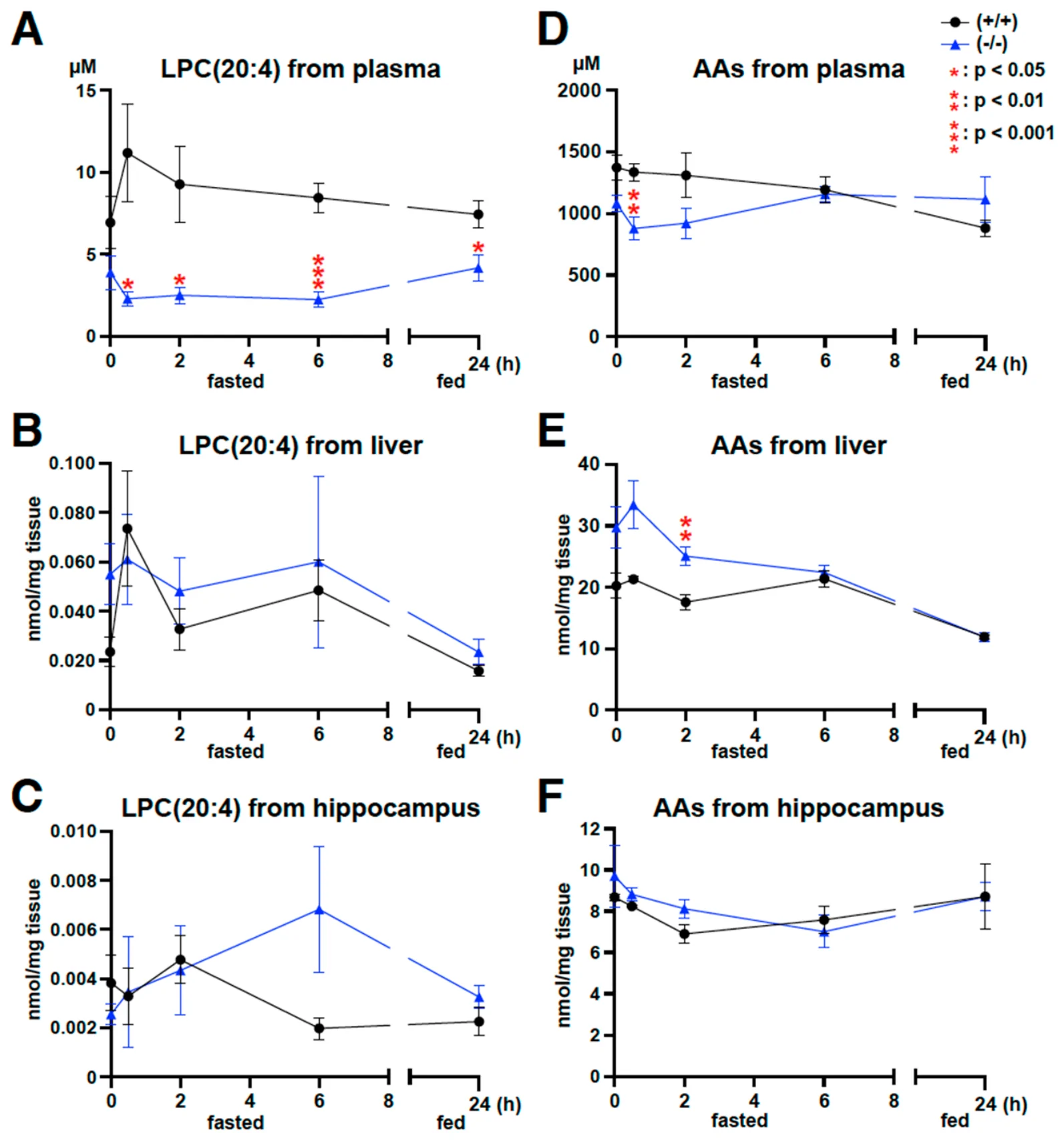
Concentration of LPC(20:4) and arachidonic acid (AA) derivatives after LPC(20:4) administration: The concentrations of LPC(20:4) in plasma (**A**), liver (**B**), and hippocampal tissue (**C**) were analyzed in *Slc22a23*^+/+^ (black circles) and *Slc22a23*^−/−^ (blue triangles) rats. The concentrations of AA derivatives obtained from all lipid classes were analyzed in plasma (**D**), liver (**E**), and hippocampal tissue (**F**). Rats (8 weeks old) were fasted for 16 h. After 16 h of fasting, plasma and tissue samples were collected from 5 *Slc22a23*^+/+^ and 5 *Slc22a23*^−/−^ rats without injection (t = 0). Subsequently, the remaining rats received LPC(20:4) at 5.437 mg (10 µmol)/kg via tail-vein, and samples were collected at 0.5 h (5 *Slc22a23*^+/+^ and 4 *Slc22a23*^−/−^ rats), 2 h (5 *Slc22a23*^+/+^ and 5 *Slc22a23*^−/−^ rats), 6 h (5 *Slc22a23*^+/+^ and 4 *Slc22a23*^−/−^ rats), and 24 h (4 *Slc22a23*^+/+^ and 4 *Slc22a23*^−/−^ rats) after LPC(20:4) administration. Plasma samples were collected from the apex of the heart. Liver (the tip of the median lobe) and hippocampus (isolated under a microscope [18]) were collected as tissue samples. For total AA analysis, fatty acids were extracted from all lipid classes, and then total AA was quantified. For tissue samples, the amounts of LPC(20:4) and AA were normalized to the initial sample weight. The error bars represent the mean ± SEM. The *p*-values indicated by asterisks (*: *p* < 0.05; **: *p* < 0.01; ***: *p* < 0.001) represent the results of the corresponding two-tailed *t*-tests. The descriptive statistics are shown in Table S3.

For the spatial and time-course metabolomics analysis, 24 *Slc22a23*^+/+^ and 22 *Slc22a23*^−/−^ male rats (8 weeks old) were used. Before intravenous LPC(20:4) administration, rats were housed individually and fasted for 16 h. To evaluate the basal LPC(20:4) and AA levels, samples were collected immediately before injection (t = 0). LPC(20:4) was then administered to the remaining rats via the tail-vein at 5.437 mg (10 µmol)/kg, and the samples were collected 0.5, 2, 6 and 24 h after administration. Plasma LPC(20:4) concentrations in *Slc22a23*^−/−^ rats were consistently lower than those in *Slc22a23*^+/+^ rats (Figure 4A). At 0.5 h after injection, mean plasma LPC(20:4) concentrations were 11.2 µM vs. 2.28 µM (*p* = 0.040); at 2 h, 9.27 µM vs. 2.49 µM (*p* = 0.042); at 6 h, 8.44 µM vs. 2.23 µM (*p* = 0.00096); at 24 h, 7.44 µM vs. 4.17 µM (*p* = 0.030), in *Slc22a23*^+/+^ rats and *Slc22a23*^−/−^ rats, respectively. However, no statistically significant difference in LPC(20:4) levels was detected in liver and hippocampal tissues (Figure 4B,C). Plasma AA derivatives were significantly higher 30 min after administration in *Slc22a23*^+/+^ rats (mean 1336.5 µM) compared to *Slc22a23*^−/−^ rats (mean 877.5 µM; *p* = 0.0078) (Figure 4D). In contrast, AA derivatives in the liver were significantly higher in *Slc22a23*^−/−^ rats (mean 25.1 nmol/mg tissue; *p* = 0.0052) than *Slc22a23*^+/+^ rats (mean 17.6 nmol/mg tissue) 2 h after administration (Figure 4E). These results suggest the existence of differences in fatty acid metabolism in the systemic circulation between *Slc22a23*^+/+^ and *Slc22a23*^−/−^ rats during fasting. However, no statistically significant differences in AA derivatives were detected in hippocampal tissue (Figure 4F), suggesting that a single intravenous LPC(20:4) administration contributes only minimally to the level of total AA content in the hippocampus and cerebral circulation. Different LPC(20:4) kinetics were observed after LPC(20:4) administration in vivo (Figure 4C). In WT rats, the average LPC(20:4) level in the hippocampus was highest at 2 h after administration, whereas in *Slc22a23* knockout rats, the level was highest at 6 h after administration. Therefore, differences between WT and *Slc22a23* knockout rats are expected in the animal behavioral experiments conducted at both 2 and 6 h after administration, as described in the following sections.

### 3.4. Open Field (OF) Test After LPC(20:4) Administration

**Figure 5:**
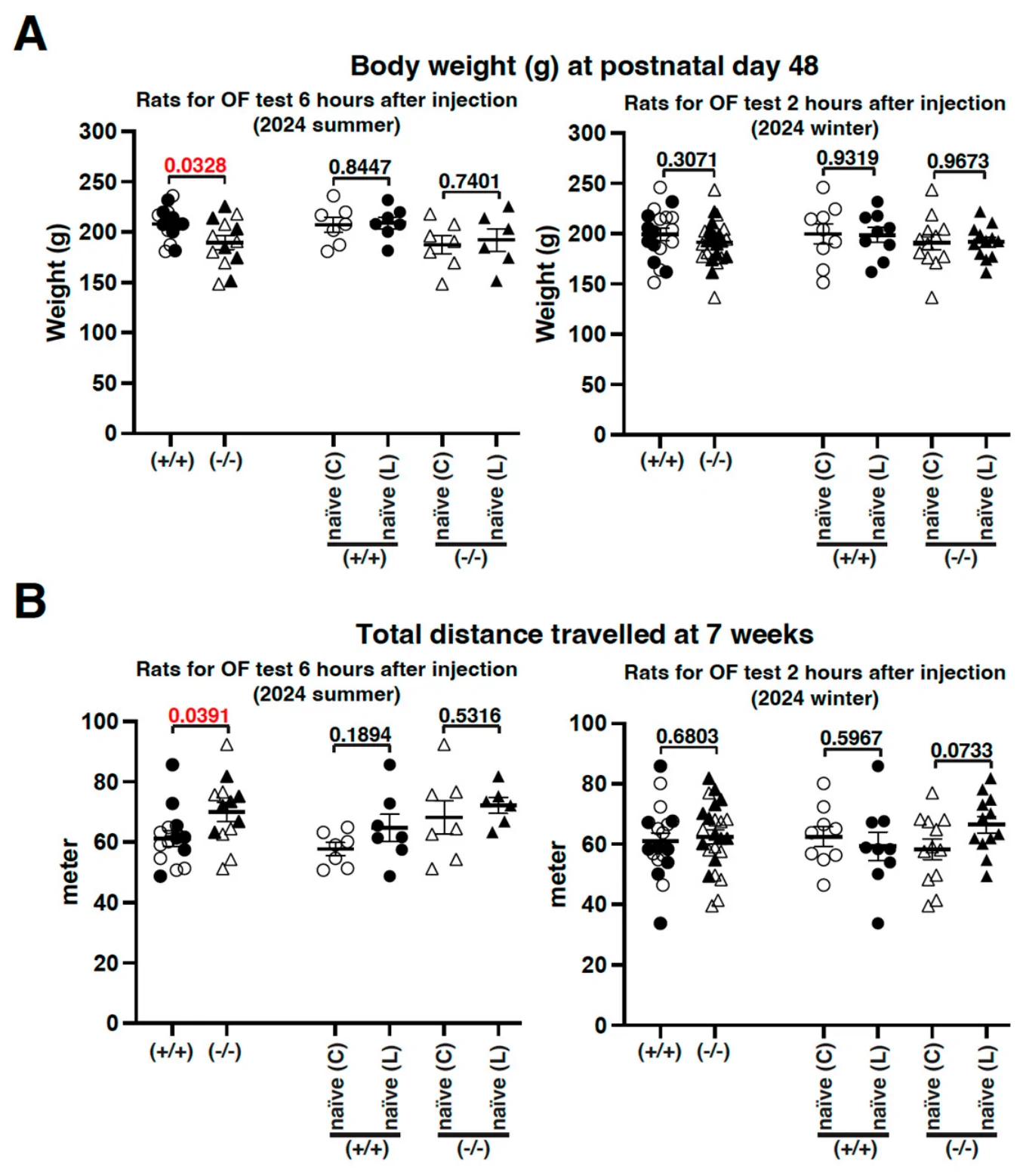
Control group (C) and LPC(20:4) injection group (L) in each genotype: (**A**) The rats were divided into two groups based on their body weight ranking on postnatal day 48 to ensure counterbalancing within each genotype. The naïve (C) are the control group, which will receive 0.9% sodium saline at 8 and 12 weeks of age. The naïve (L) are the LPC(20:4) injection group, which will receive a dose of 5.437 mg (10 µmol)/kg of LPC(20:4) at 8 and 12 weeks of age. In the 2024 summer experiment, the exact sample sizes were *Slc22a23*^+/+^ control (*n* = 7), *Slc22a23*^+/+^ with LPC(20:4) injection (*n* = 7), *Slc22a23*^−/−^ control (*n* = 7), and *Slc22a23*^−/−^ with LPC(20:4) injection (*n* = 6), respectively. In the 2024 winter experiment, the exact sample sizes were *Slc22a23*^+/+^ control (*n* = 9), *Slc22a23*^+/+^ with LPC(20:4) injection (*n* = 9), *Slc22a23*^−/−^ control (*n* = 12), and *Slc22a23*^−/−^ with LPC(20:4) injection (*n* = 12), respectively. (**B**) Total distance travelled in the OF test at 7 weeks of age. Each naïve rat (with no history of injections or treatments) was placed in a circular chamber (100 cm diameter, 45 cm height), and movements of more than 1 cm were recorded for 10 min. The error bars represent the mean ± SEM. The *p*-value shown above each square bracket represents the result of the corresponding two-tailed *t*-test. The descriptive statistics are shown in Table S3.

For the OF test, we used *Slc22a23*^+/+^ and *Slc22a23*^−/−^ rats. As previously described [4], the body weight of *Slc22a23*^−/−^ rats was statistically significantly lower than that of *Slc22a23*^+/+^ rats (average 189.6 g and 208.2 g, respectively, in the experiment 2024 summer; *p*-value = 0.0328). Before conducting the OF test, rats of each genotype were divided into two groups: an LPC(20:4) administration group and a saline administration group. To allocate the animals, these rats were divided based on their body weight ranking in each genotype. Within each genotype, the test rats were divided at postnatal day 48 into two groups: naïve (C) and naïve (L) (Figure 5A). Here, “naïve” refers to rats that have not yet received any injections, either LPC(20:4) or sodium saline. At 8 and 12 weeks of age, the naïve (C) rats will receive 0.9% sodium saline as the “Control” treatment, while the naïve (L) rats will receive a dose of 5.437 mg (10 µmol)/kg of LPC(20:4) (Figure 1A,B), as well as at 9 and 13 weeks of age for social interaction test (Figure 1A,B). Their total distance travelled at 7 weeks of age was measured in the OF. The naïve *Slc22a23*^−/−^ rats showed statistically significantly increased spontaneous exploratory movements compared to those of *Slc22a23*^+/+^ rats (average 70.0 m in *Slc22a23*^−/−^ and average 61.2 m in *Slc22a23*^+/+^, respectively, with a *p*-value of 0.0391) (Figure 5B), as previously described [4]. The two groups, the naïve (C) and naïve (L), divided based on their body weight ranking within each genotype, showed no statistically significant difference in total distance travelled in the OF test at 7 weeks of age (Figure 5B). Since we had previously compared the two genotypes (naïve *Slc22a23*^+/+^ and naïve *Slc22a23*^−/−^) in our earlier study [4], we focused this study specifically on evaluating the effects of LPC(20:4) administration within each genotype.

**Figure 6:**
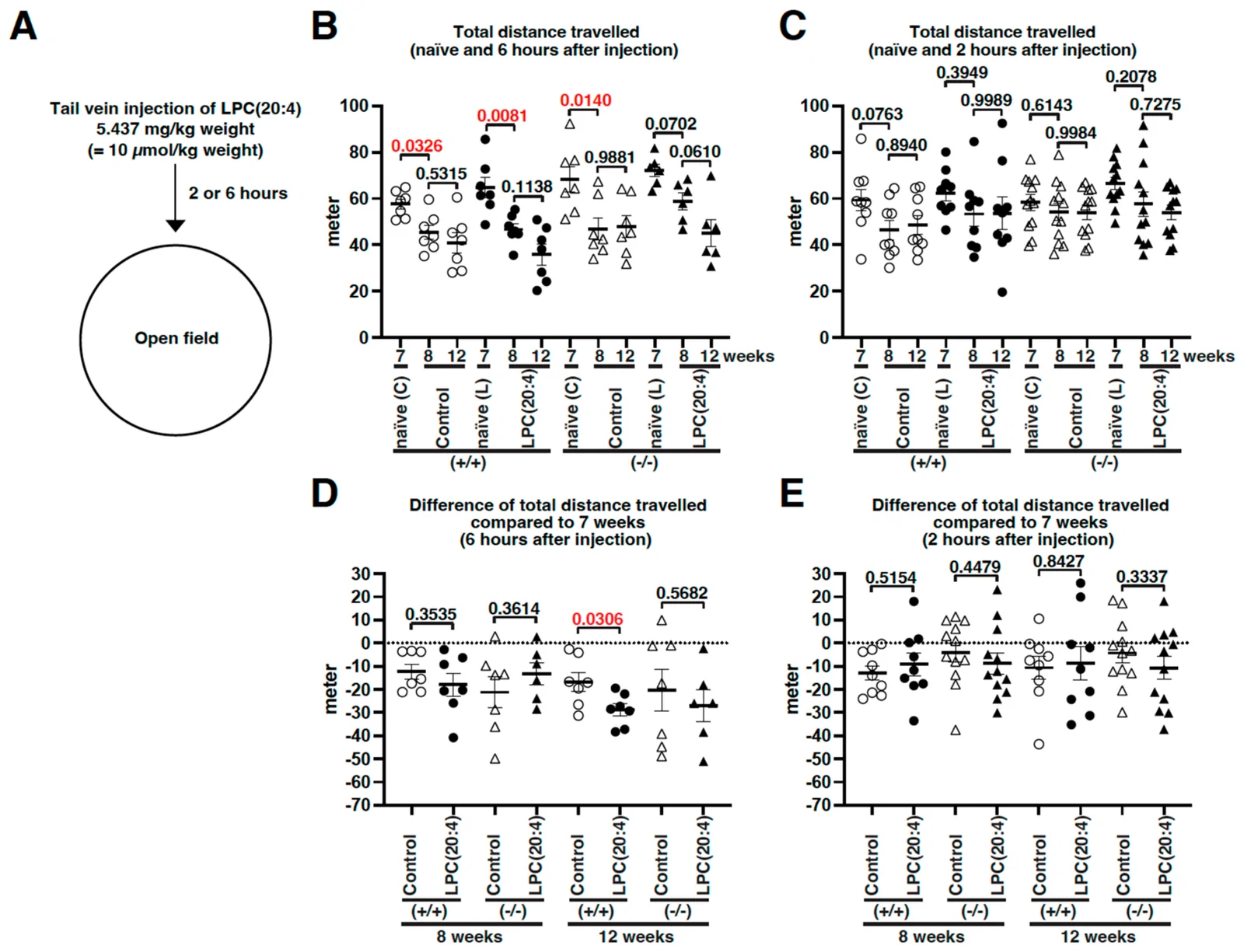
Spontaneous movements after LPC(20:4) administration: (**A**) The scheme of the OF test. In the 2024 summer experiment (6 h after injection), the exact sample sizes were *Slc22a23*^+/+^ control (*n* = 7), *Slc22a23*^+/+^ with LPC(20:4) injection (*n* = 7), *Slc22a23*^−/−^ control (*n* = 7), and *Slc22a23*^−/−^ with LPC(20:4) injection (*n* = 6), respectively. In the 2024 winter experiment (2 h after injection), the exact sample sizes were *Slc22a23*^+/+^ control (*n* = 9), *Slc22a23*^+/+^ with LPC(20:4) injection (*n* = 9), *Slc22a23*^−/−^ control (*n* = 12), and *Slc22a23*^−/−^ with LPC(20:4) injection (*n* = 12), respectively. (**B**,**C**) The total distance travelled of the naïve rats at 7 weeks of age, as well as 6 h after injection at 8 and 12 weeks of age (**B**), or 2 h after injection at 8 and 12 weeks of age (**C**). The error bars represent the mean ± SEM. To examine cumulative effects in the time-series experiment, values at 7 weeks were compared with those at 8 weeks, and values at 12 weeks were compared with those at 8 weeks. The *p*-value shown above each square bracket represents the result of the corresponding Dunnett’s multiple comparisons. (**D**,**E**) The difference in total distance travelled between 7 and 8 weeks of age, or between 7 and 12 weeks of age. The total distance travelled at 7 weeks of age was subtracted from the total distance travelled at 8 weeks of age or 12 weeks of age ((**D**): 6 h after injection, (**E**): 2 h after injection). The error bars represent the mean ± SEM. The *p*-value shown above each square bracket represents the result of the corresponding two-tailed *t*-test. The descriptive statistics are shown in Table S3.

At 8 and 12 weeks of age, the test rats received a dose of 5.437 mg (10 µmol)/kg of LPC(20:4) or 0.9% sodium saline via tail-vein either 6 h (2024 summer) or 2 h (2024 winter) before the OF test (Figure 6A). Since maturation (from adolescence or juvenile stages to full adulthood) and repeated experiences typically decrease the total distance travelled in the OF, the total distance travelled at 8 weeks of age was statistically significantly reduced, regardless of their genotype, compared to naïve rats at 7 weeks of age (Figure 6B). On the other hand, the total distance travelled in the OF test conducted 2 h after administration (2024 winter) showed no statistically significant difference (Figure 6C). At 12 weeks of age, the reductions in total distance travelled relative to 7 weeks of age were statistically significantly different between the LPC(20:4) administered group and the control group in the *Slc22a23*-proficient rats (LPC(20:4): an average decrease of 28.9 m; control: an average decrease of 16.8 m; *p*-value = 0.0306) (Figure 6D). In contrast, the reductions in *Slc22a23*-deficient rats were not statistically significantly different (Figure 6D). However, when the OF test was conducted 2 h after administration, the reductions relative to 7 weeks of age were not statistically significant in either *Slc22a23*-proficient or *Slc22a23*-deficient rats (Figure 6E).

### 3.5. Novel Object Recognition (NOR) Test After LPC(20:4) Administration

**Figure 7:**
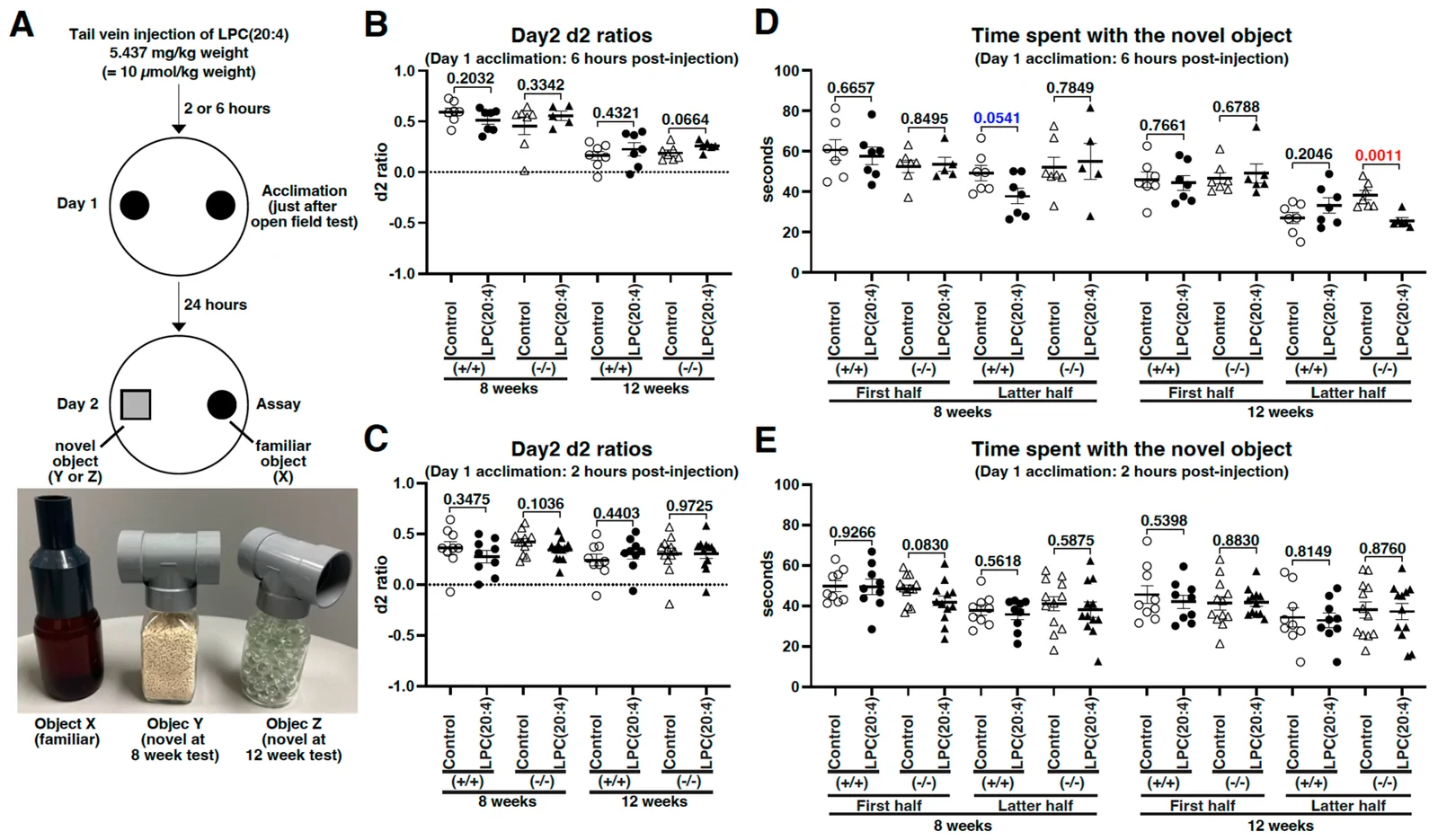
Novel object recognition (NOR) after LPC(20:4) administration: (**A**) The scheme and the objects of the NOR test. In the 2024 summer experiment (6 h after injection), the exact sample sizes were *Slc22a23*^+/+^ control (*n* = 7), *Slc22a23*^+/+^ with LPC(20:4) injection (*n* = 7), *Slc22a23*^−/−^ control (*n* = 7), and *Slc22a23*^−/−^ with LPC(20:4) injection (*n* = 6), respectively. In the 2024 winter experiment (2 h after injection), the exact sample sizes were *Slc22a23*^+/+^ control (*n* = 9), *Slc22a23*^+/+^ with LPC(20:4) injection (*n* = 9), *Slc22a23*^−/−^ control (*n* = 12), and *Slc22a23*^−/−^ with LPC(20:4) injection (*n* = 12), respectively. (**B**,**C**) Discrimination (d2) ratios in the novel object recognition test on day 2. The d2 ratio was calculated using the following formula: d2 = [time(novel object) − time(familiar object)]/time(novel object + familiar object) [19]. Acclimations to Object X on day 1 were conducted 6 h after injection (**B**), 2 h after injection (**C**), at 8 and 12 weeks of age. The error bars represent the mean ± SEM. The *p*-value shown above each square bracket represents the result of the corresponding two-tailed *t*-test. (**D**,**E**) Comparisons of the duration of access to the novel object during the first half (0–5 min) and the latter half (5–10 min) of the NOR test. Access was defined as the time when the head of a test rat was within 7 cm of the wall of the object. The error bars represent the mean ± SEM. The *p*-value shown above each square bracket represents the result of the corresponding two-tailed *t*-test. The descriptive statistics are shown in Table S3.

The caps of the novel objects used on day 2 in the NOR test had two large holes, one on each side (Figure 7A). The novel objects attracted the test rats much more strongly than the novel objects in the previous study [4], resulting in highly positive d2 ratios (Figure 7B,C), and this is a potential caveat of this assay. Therefore, we simply compared the duration of access to the novel object, defined as the time when the head of a test rat was within 7 cm of the wall of the object. The duration of access to the novel object during the first half (0–5 min) was not statistically significantly different between the control and the LPC(20:4) administered groups, regardless of genotype and injection time (Figure 7D,E). On the other hand, in the NOR test in which the day 1 acclimation was conducted 6 h after injection, the duration of access to the novel object during the latter half (5–10 min) was notably shorter in the LPC(20:4) administered *Slc22a23*^+/+^ rats at 8 weeks of age (LPC(20:4) group: average 37.8 s; control group: average 49.2 s; *p*-value = 0.0541) (Figure 7D). Even more strikingly, the duration was statistically significantly shorter in LPC(20:4) administered *Slc22a23*^−/−^ rats at 12 weeks of age (LPC(20:4) group: average 25.7 s; control group: average 38.2 s; *p*-value = 0.0011) (Figure 7D). However, no statistically significant differences were observed in the duration of access to the novel object during the latter half (5–10 min) in the NOR test, in which the day 1 acclimation was conducted 2 h after injection (Figure 7E).

### 3.6. Social Interaction (SI) Test After LPC(20:4) Administration

**Figure 8:**
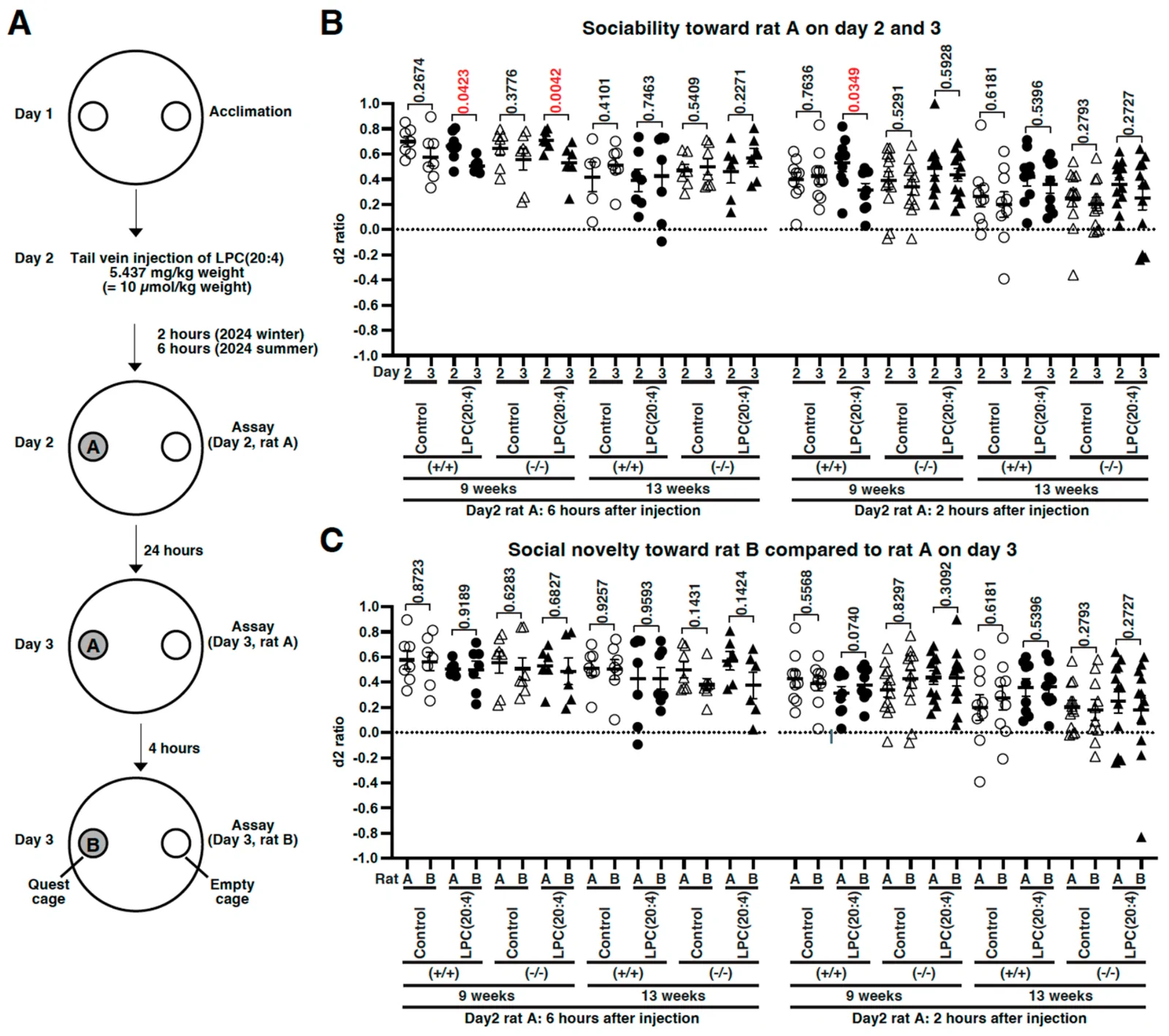
Social interaction (SI) with intruder rats after LPC(20:4) administration: (**A**) The scheme of the SI test. In the 2024 summer experiment (6 h after injection), the exact sample sizes were *Slc22a23*^+/+^ control (*n* = 7), *Slc22a23*^+/+^ with LPC(20:4) injection (*n* = 7), *Slc22a23*^−/−^ control (*n* = 7), and *Slc22a23*^−/−^ with LPC(20:4) injection (*n* = 6), respectively. In the 2024 winter experiment (2 h after injection), the exact sample sizes were *Slc22a23*^+/+^ control (*n* = 9), *Slc22a23*^+/+^ with LPC(20:4) injection (*n* = 9), *Slc22a23*^−/−^ control (*n* = 12), and *Slc22a23*^−/−^ with LPC(20:4) injection (*n* = 12), respectively. (**B**) Comparison of sociability toward rat A on day 2 and day 3. The discrimination ratio (d2) was calculated using the following formula: d2 = [time(quest cage) − time(empty cage)]/time(quest cage + empty cage). Rat A was in the quest cage on day 2 and day 3. (**C**) Comparisons of social novelty toward rat B compared to rat A on day 3. After the SI test using rat A, the same analysis was conducted using rat B in the quest cage. The d2 ratios in the first half of the test (5 min) were compared. The error bars represent the mean ± SEM. The *p*-value shown above each square bracket represents the result of the corresponding paired two-tailed *t*-tests. The descriptive statistics are shown in Table S3.

At 9 and 13 weeks of age, SI tests using two intruder rats (rat A as a familiar rat, while rat B as a novel rat) were conducted (Figure 8A). The LPC(20:4) injection, but not the saline injection, produced a statistically significant difference in sociability toward rat A between day 2 and day 3 at 9 weeks of age (Figure 8B). The d2 ratio decreased from an average of 0.66 on day 2 to 0.51 on day 3, with a *p*-value of 0.0423, in the *Slc22a23*^+/+^, and from an average of 0.71 on day 2 to 0.53 on day 3, with a *p*-value of 0.0042, in the *Slc22a23*^−/−^. Even in the SI test conducted 2 h after LPC(20:4) injection, *Slc22a23*^+/+^ rats exhibited a statistically significant difference in sociability toward rat A between day 2 and day 3 at 9 weeks of age (Figure 8B). The d2 ratio was decreased from an average of 0.53 on day 2 to 0.31 on day 3, with a *p*-value of 0.0349. However, no statistically significant difference was observed in our social novelty assay toward rat B (the novel rat) compared to rat A (the familiar rat) when rat B was presented 4 h after rat A on day 3 (Figure 8C). Social novelty might show differences when the familiar rat and the stranger rat were presented simultaneously [25].

### 3.7. Morris Water Maze (MWM) Test After LPC(20:4) Administration

**Figure 9:**
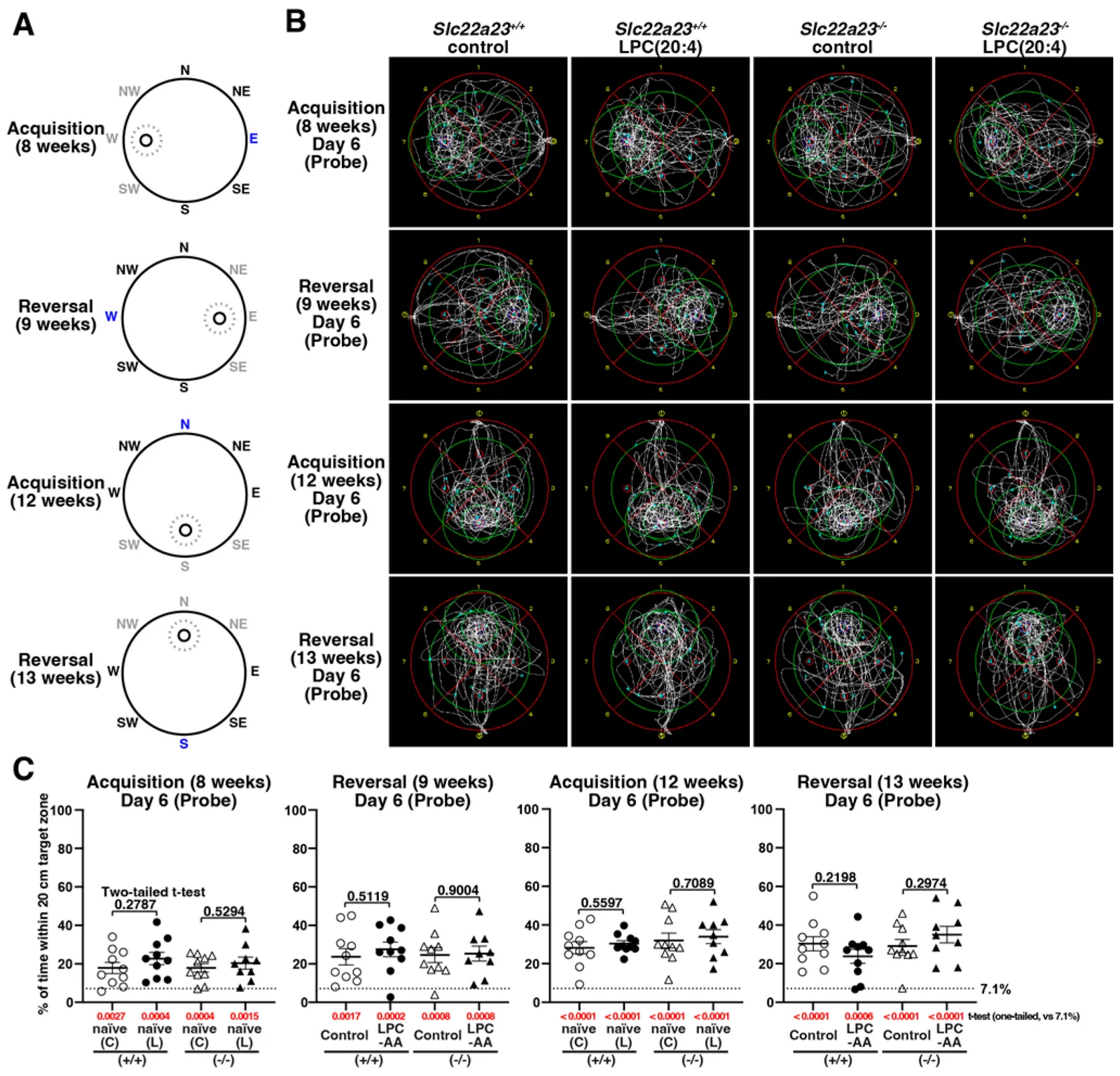
The scheme and the results of probe sessions in the Morris water maze (MWM) test: (**A**) The scheme of the MWM test. A black platform was placed in the west (W) at 8 weeks of age, in the east (E) at 9 weeks of age, in the south (S) at 12 weeks of age, and in the north (N) at 13 weeks of age, respectively, with the center of the platform positioned 30 cm away from the wall. The test rats were released from each position (listed in Table 1) facing the wall. In the MWM test, the exact sample sizes were *Slc22a23*^+/+^ control (*n* = 10), *Slc22a23*^+/+^ with LPC(20:4) injection (*n* = 10), *Slc22a23*^−/−^ control (*n* = 10), and *Slc22a23*^−/−^ with LPC(20:4) injection (*n* = 9), respectively. (**B**) Combined trajectories of the 30-s probe sessions (without platform) in each group. Each test rat was released from the opposite side of the pool, facing the wall (shown in blue in Figure 9A). (**C**) Percentage of time spent within 20 cm from the center of the target site. The error bars represent the mean ± SEM. The *p*-value shown above each square bracket represents the result of the corresponding paired two-tailed *t*-tests comparing within each genotype. The results of one-tailed *t*-tests against 7.1%, presenting the proportion of the circle area (radius 20 cm) relative to the total pool surface, are shown below the graph. The descriptive statistics are shown in Table S3.

To further investigate the effects of LPC(20:4) administration, particularly on spatial memory acquisition, we conducted an MWM test. During the 5-consecutive-days of trials (Table 1), a black platform was placed in the west (W), east (E), south (S), and north (N) positions at 8, 9, 12, and 13 weeks of age, respectively (Figure 9A). On the 6th day in each acquisition and reversal phase (Table 1), 30-s probe sessions (without platform) were conducted. Each test rat was released from the opposite side of the pool, facing the wall. Trajectories of the 30-s probe sessions were plotted and combined for each group (Figure 9B). The duration of the test rats spent within 20 cm of the center of the target site was plotted (Figure 9C). The area within 20 cm corresponds to 7.1% of the total surface area of the pool. The results showed that, regardless of genotype or LPC(20:4) administration, the test rats preferred to stay within 20 cm of the area statistically significantly during each 30-s probe session (Figure 9C).

**Figure 10:**
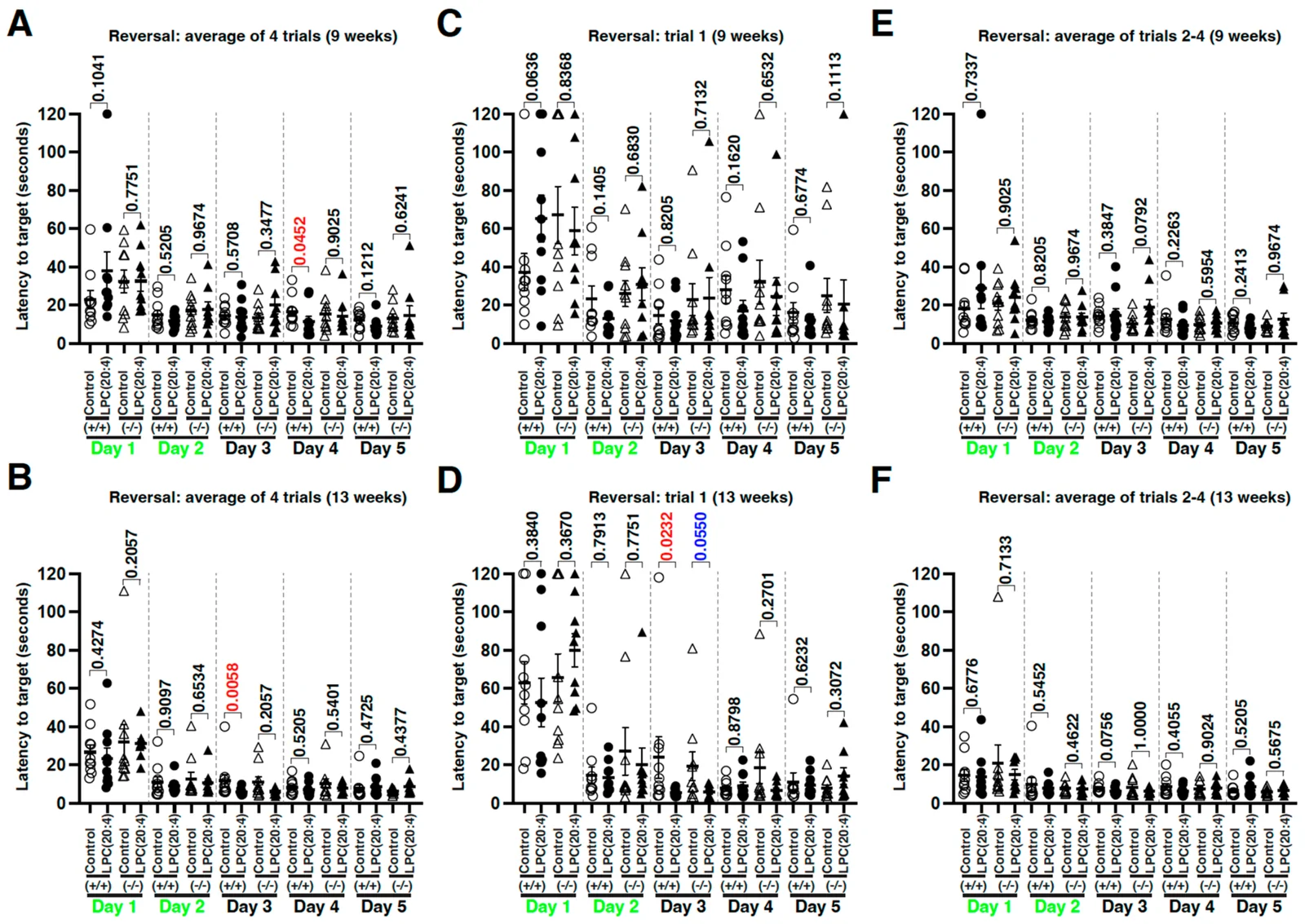
Latency to the target after LPC(20:4) administration in the reversal phase of the MWM test: (**A**,**B**) Average latency to the target (seconds) of 4 trials during the reversal phase ((**A**): 9 weeks of age, (**B**): 13 weeks of age). (**C**,**D**) Latency to the target (seconds) for the first trial in each day during the reversal phase ((**C**): 9 weeks of age, (**D**): 13 weeks of age). (**E**,**F**) Average latency to the target (seconds) of trials 2, 3, and 4 in each day during the reversal phase ((**E**): 9 weeks of age, (**F**): 13 weeks of age). A dose of 5.437 mg (10 µmol)/kg of LPC(20:4) or 0.9% sodium saline was injected intravenously via tail-vein into the test rats 6 h before the MWM test on days 1 and 2 of the reversal phase. In the MWM test, the exact sample sizes were *Slc22a23*^+/+^ control (*n* = 10), *Slc22a23*^+/+^ with LPC(20:4) injection (*n* = 10), *Slc22a23*^−/−^ control (*n* = 10), and *Slc22a23*^−/−^ with LPC(20:4) injection (*n* = 9), respectively. The descriptive statistics are shown in Table S3. Because of the skewed distributions of the latency to the target (seconds), the *p*-value shown above each square bracket was calculated with a nonparametric Wilcoxon/Kruskal–Wallis test (rank sums, Wilcoxon two-sample test, normal approximation). The error bars represent the mean ± SEM. The descriptive statistics are shown in Table S3. The Latencies to the target in the acquisition phase of the corresponding MWM tests are shown in Figure S1.

Day 1 of the reversal phase was conducted 24 h after the acquisition probe session on day 6. To evaluate the effect of LPC(20:4) administration on the relearning of spatial memory (cognitive flexibility), the test rats received a dose of 5.437 mg (10 µmol)/kg of LPC(20:4) or 0.9% sodium saline intravenously via tail-vein 6 h before the MWM test on days 1 and 2 of the reversal phase (Table 1). Because the more pronounced differences were observed in the animal behavioral tests (OF, NOR, and SI) conducted 6 h after injection (2024 summer) than those conducted 2 h after injection (2024 winter) (Figure 6, Figure 7 and Figure 8), suggesting potential pharmacokinetic differences, we decided to conduct the MWM test 6 h after injection (Figure 1C, green arrows (2025 spring)). The latency to the target (seconds) exhibited substantially skewed distributions, with skewness values exceeding 2.0 (Figure 10, Table S3). It is generally proposed that a skewness greater than 2.0 (or less than −2.0) indicates a substantial departure from a symmetrical distribution [26]. Therefore, we analyzed the latencies to the target (seconds) using a nonparametric Wilcoxon/Kruskal–Wallis test (rank sums, Wilcoxon two-sample test, normal approximation). In *Slc22a23*^+/+^ rats, the LPC(20:4) administration, but not the saline administration, caused a statistically significantly shorter latency to the target on day 4 at 9 weeks of age (LPC(20:4): average 11.4 s; control: average 16.5 s; *p*-value = 0.0452) (Figure 10A) and on day 3 at 13 weeks of age (LPC(20:4): average 5.9 s; control: average 11.8 s; *p*-value = 0.0058) (Figure 10B). We then analyzed trial 1 and trials 2–4 separately (Figure 10C–F). Trial 1 was conducted 24 h after the previous trial. Therefore, its latency to the target was assumed to reflect long-term memory. Trials 2–4 were conducted at 15-s intervals. Therefore, their latencies to the target were assumed to reflect short-term memory. Although no statistically significant differences were observed in the first trial of 9 weeks of age (Figure 10C), the latencies to the target at the trial 1 on day 3 of the 13-week-old *Slc22a23*^+/+^ rats were statistically significantly shorter in LPC(20:4) administered group compared with the control group (LPC(20:4): average 5.3 s; control: average 24.0 s, *p*-value = 0.0232) (Figure 10D). Also, a notable difference was observed in trial 1 on day 3 of the 13-week-old *Slc22a23*^−/−^ rats (LPC(20:4): average 5.7 s; control: average 19.3 s, *p*-value = 0.0550) (Figure 10D). However, no statistically significant differences were observed in the average latencies to the target in trials 2, 3, and 4 on each day (Figure 10E,F). These results suggest that LPC(20:4) administration facilitates the acquisition of long-term memory and the relearning of spatial memory, particularly in *Slc22a23*-proficient WT rats, but also, to some extent, in *Slc22a23*-deficient knockout rats. Further studies are needed to confirm these findings.

**Figure 11:**
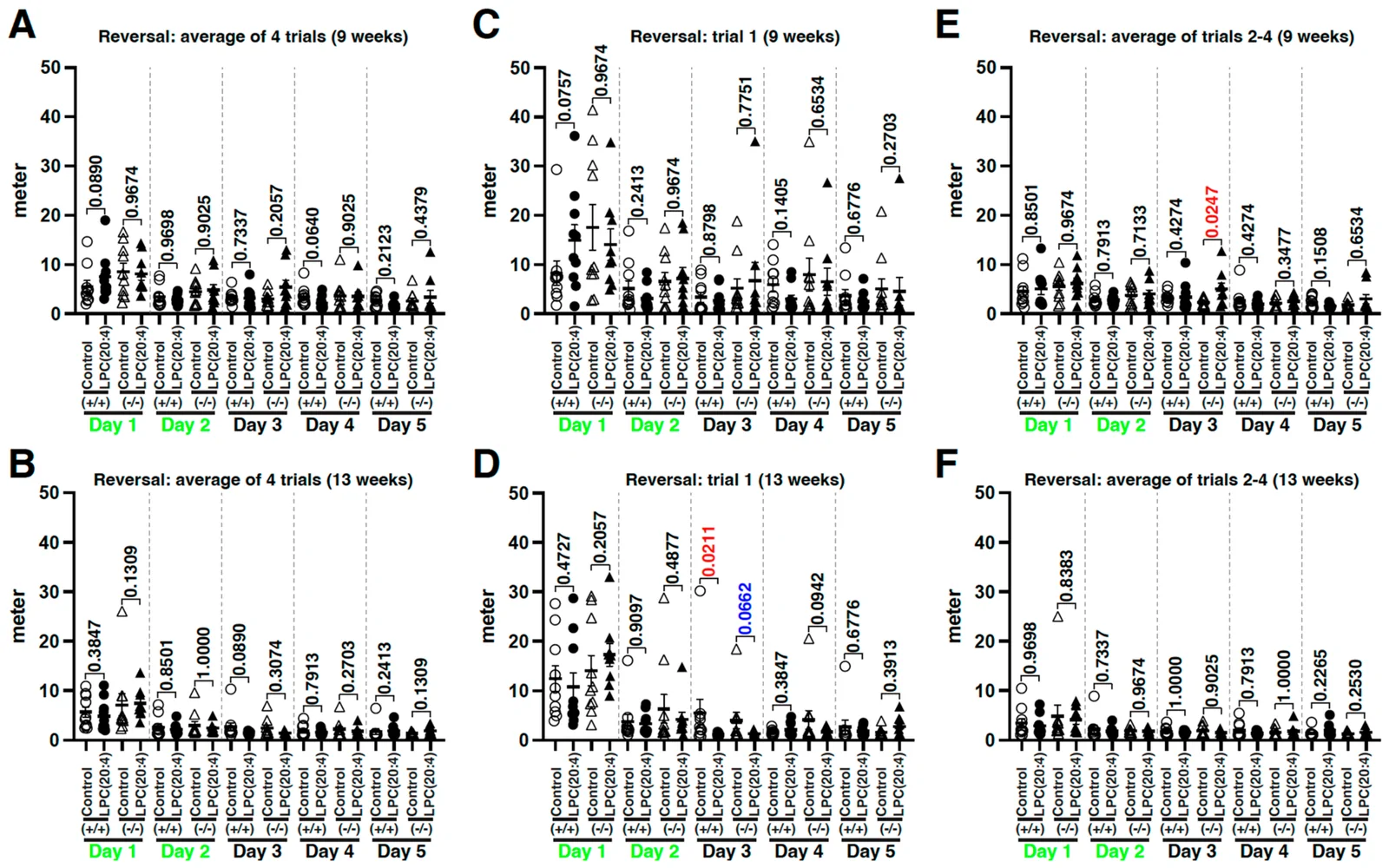
Swim distance from start to goal. Using the same MWM dataset as in Figure 10, but the swim distances (meters) from start to goal were plotted. (**A**,**B**) Swim distance (meters) of 4 trials during the reversal phase ((**A**): 9 weeks of age, (**B**): 13 weeks of age); (**C**,**D**) Swim distance (meters) for the first trial in each day during the reversal phase ((**C**): 9 weeks of age, (**D**): 13 weeks of age); (**E**,**F**) Swim distance (meters) of trials 2, 3, and 4 in each day during the reversal phase ((**E**): 9 weeks of age, (**F**): 13 weeks of age). The test rats (Wistar; white coat color) occasionally dove under the water in the pool, which was stained with a black watercolor paint. During these periods, the video tracking system (CompACT video tracking system, Muromachi Kikai) could not track their movement, and the trajectory could not be recorded reliably. The shortest possible distance from the start point to the goal was 83 cm. Therefore, the swim distance of less than 70 cm was excluded as measurement errors. Because of the skewed distributions of the swim distance, the *p*-value shown above each square bracket was calculated with a nonparametric Wilcoxon/Kruskal–Wallis test (rank sums, Wilcoxon two-sample test, normal approximation). The error bars represent the mean ± SEM. The descriptive statistics are shown in Table S3. The swim distances from start to goal in the acquisition phase of the corresponding MWM tests are shown in Figure S2.

Using the same MWM dataset, swim distances (meters) from start to goal were examined (Figure 11). Consistent with the shorter latency to the target at the trial 1 on day 3 of the 13-week-old *Slc22a23*^+/+^ rats by LPC(20:4) administration (Figure 10D), the average swim distance of WT rats was also significantly shorter in the LPC(20:4) administered group compared to that of the control group (control group: average 5.46 m; LPC(20:4) group: average 1.14 m; *p*-value = 0.0211) (Figure 11D). Similarly, the average swim distance of *Slc22a23* knockout rats was notably shorter at trial 1 on day 3 of the 13-week-old *Slc22a23*^−/−^ rats in the LPC(20:4) administered group compared to that of the control group (control group: average 3.99 m; LPC(20:4) group: average 1.30 m; *p*-value = 0.0662) (Figure 11D). In contrast, at 9 weeks of age, a significantly longer swim distance was observed in LPC(20:4) administered *Slc22a23* knockout rats (control group: average 2.27 m; LPC(20:4) group: average 5.01 m; *p*-value = 0.0247) (Figure 11E).

**Figure 12:**
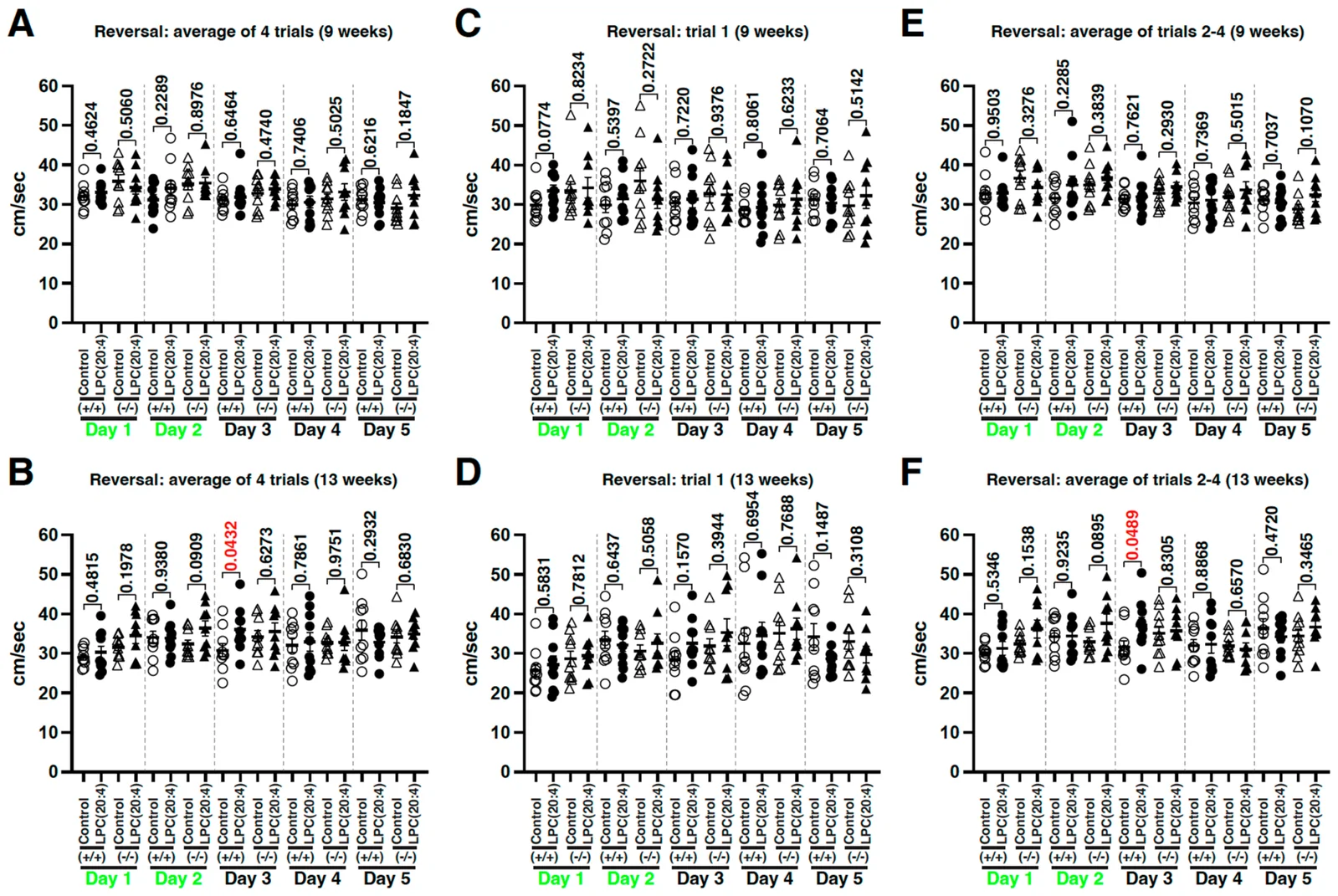
Swim speed from start to goal. Using the same MWM dataset as in Figure 10, the swim speeds (cm/s) from start to goal were plotted. (**A**,**B**) Swim speeds (cm/s) of 4 trials during the reversal phase ((**A**) 9 weeks of age, (**B**): 13 weeks of age); (**C**,**D**) Swim speeds (cm/s) for the first trial in each day during the reversal phase ((**C**): 9 weeks of age, (**D**): 13 weeks of age); (**E**,**F**) Swim speeds (cm/s) of trials 2, 3, and 4 in each day during the reversal phase ((**E**): 9 weeks of age, (**F**): 13 weeks of age). As noted in the figure legend for Figure 11, the test rats (Wistar; white coat color) occasionally dove under the water in the pool, which was stained with a black watercolor paint. During these periods, the video tracking system (CompACT video tracking system, Muromachi Kikai) could not track their movement, and the trajectory could not be recorded reliably. In MWM tests, adult Wistar rats typically swim at an average speed of 20–40 cm/s [27,28]. Therefore, swim speeds greater than 60 cm/s were excluded as measurement errors. The *p*-value shown above each square bracket represents the result of the corresponding two-tailed *t*-test. The error bars represent the mean ± SEM. The descriptive statistics are shown in Table S3. The swim speeds from start to goal in the acquisition phase of the corresponding MWM tests are shown in Figure S3.

Using the same MWM dataset, average swim speeds (cm/s) from start to goal were examined (Figure 12). Consistent with the shorter latency to the target of average 4 trials of WT rats by LPC(20:4) administration on day 3 at 13 weeks of age (Figure 10B), swim speed was significantly higher in LPC(20:4) administered WT rats than those in the saline administered WT rats on day 3 at 13 weeks of age (LPC(20:4): average 36.0 cm/s; control: average 30.8 cm/s; *p*-value = 0.0432) (Figure 12B). This difference most likely arose from the swim speed in trials 2–4 on the day, because, although no statistically significant differences were observed in the first trial (Figure 12D), the average swim speed in trials 2–4 on day 3 was significantly higher in LPC(20:4) administered WT rats than in the saline administered WT rats (LPC(20:4): average 37.1 cm/s; control: average 31.6 cm/s; *p*-value = 0.0489) (Figure 12F).

### 3.8. LPC(20:4) Incorporation Efficiency in Rat Primary Hippocampal Neurons

**Figure 13:**
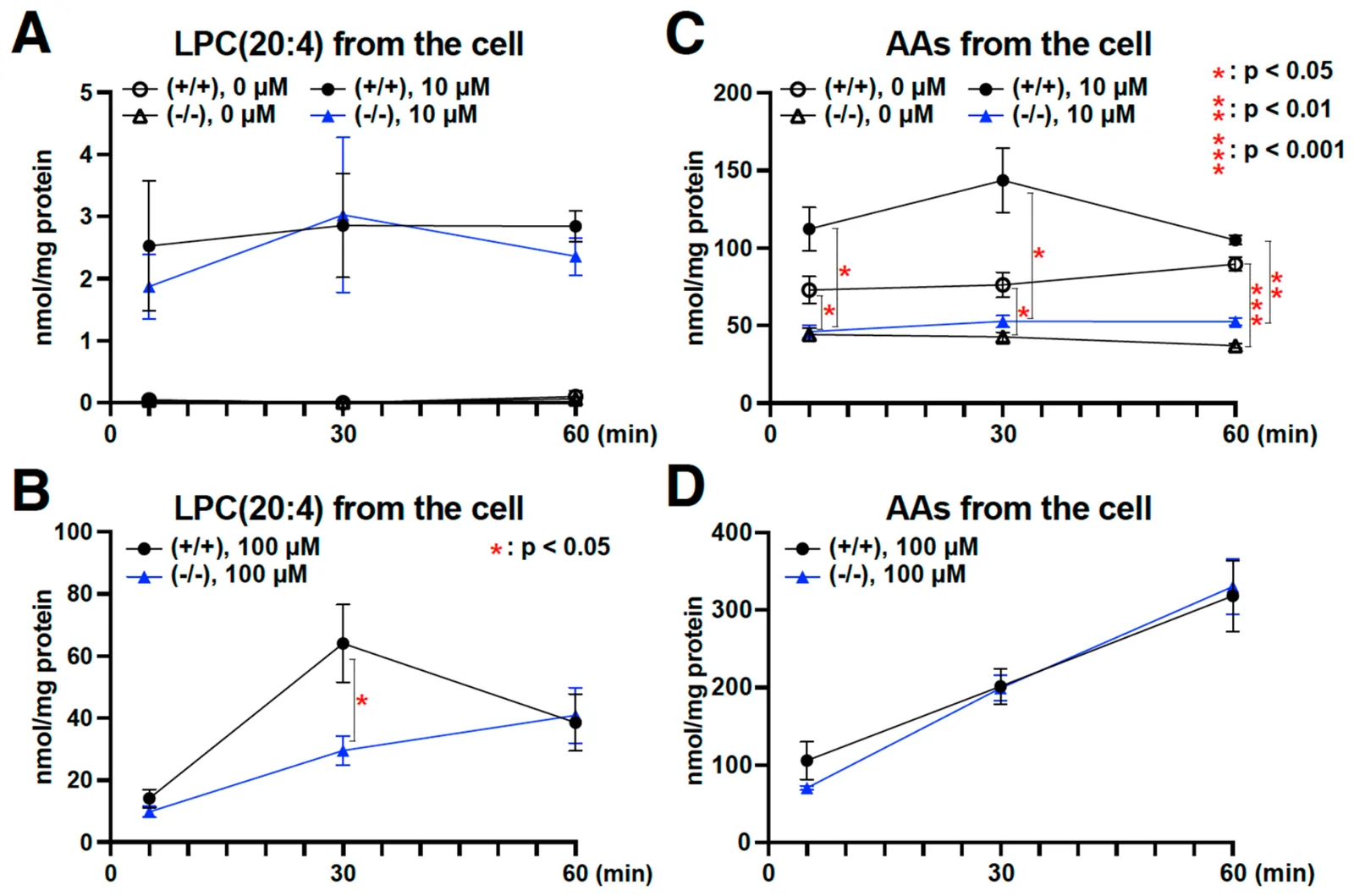
LPC(20:4) incorporation assay using rat primary hippocampal neurons: The concentrations of LPC(20:4) with/within cells were measured after treatment with 0 or 10 µM LPC(20:4) (**A**) and 100 µM LPC(20:4) (**B**). The concentrations of AA derivatives obtained from all lipid classes were measured after treatment with 0 or 10 µM LPC(20:4) (**C**) and 100 µM LPC(20:4) (**D**). Primary hippocampal neurons were prepared from *Slc22a23*^+/+^ (WT; black circles) and *Slc22a23*^−/−^ (KO; blue triangles) rats. After culturing for 7 days, cells were washed once with D-PBS(+). Then, 2 mL of D-PBS(+) alone, 2 mL of 10 µM LPC(20:4) in D-PBS(+) (**A**,**C**), or 2 mL of 100 µM LPC(20:4) in D-PBS(+) (**B**,**D**) was added to the cell culture in each 35-mm dish. After incubation at 37 °C for 5, 30, or 60 min, cells were washed twice with D-PBS(+), 1 mL of 100% methanol was added to the cells, and the cells were scraped and stored at −80 °C until mass spectrometry analysis. The error bars represent the mean ± SEM. The *p*-values indicated by asterisks (*: *p* < 0.05; **: *p* < 0.01; ***: *p* < 0.001) represent the results of the corresponding two-tailed *t*-tests. The descriptive statistics are shown in Table S3.

Primary hippocampal neurons derived from *Slc22a23^+/+^* (WT) and *Slc22a23^−/−^* (KO) rats were used to evaluate LPC(20:4) incorporation. Before the incorporation experiment, the cells were washed once with D-PBS(+). When LPC(20:4) dissolved in D-PBS(+) was added to the cell culture at a concentration of 10 µM, no significant differences were observed between WT and KO neurons (Figure 13A). However, when the LPC(20:4) concentration was increased 10-fold, a substantial and significant difference was detected 30 min after incubation (Figure 13B). LPC(20:4) with/within the cells was an average 64.0 nmol/mg protein in WT neurons and an average 29.6 nmol/mg protein in KO neurons (*p* = 0.049). Although 100 µM is higher than physiological LPC(20:4) concentrations, the higher LPC(20:4) level with/within WT cells suggests that SLC22A23 can facilitate LPC(20:4) binding or uptake. In contrast, the amounts of AA derivatives were significantly and consistently lower in KO cells than in WT cells under both D-PBS(+) alone and 10 µM LPC(20:4) incubation (Figure 13C). On the other hand, no significant difference in AA levels was detected with 100 µM LPC(20:4) incubation (Figure 13D), likely because the measured AA derivatives include AA species derived from the excess LPC(20:4) added.

## 4. Discussion

### 4.1. Identification of LPC(20:4) as a Candidate Substrate of SLC22A23 Transporter

Mass spectrometry analysis identified 8 phospholipids with significantly lower levels in the plasma of *Slc22a23* knockout rats compared with the plasma of *Slc22a23*^+/+^ rats. All of the identified phospholipids potentially contain long-chain polyunsaturated fatty acids (LC-PUFAs) as one of their fatty acid tail(s). These 8 phospholipids include two lysophosphatidylcholines: LPC(20:4) and LPC(18:2) (Figure 2A,B). Based on the reported fatty acid composition in the plasma of male Wistar rats—specifically, an eicosatetraenoic acid [ETA; C20:4n3] to arachidonic acid [AA; C20:4n6] ratio = 1:169.7 [29]—the LPC a 20:4 detected by this mass spectrometry analysis most likely contained a single chain of AA esterified to glycerophosphocholine (GPC), rather than an ETA chain. Likewise, LPC a 18:2 is a monoacyl GPC, which must have a single chain of linoleic acid (C18:2n6) esterified to GPC. Linoleic acid is an essential fatty acid and a precursor of AA synthesis. Among the identified 8 phospholipids, PC aa C34:4, PC aa C36:4, PC ae C36:4, and PC ae C40:4 may correspond to PC (14:0/20:4), PC (16:0/20:4), PC (O-16:0/20:4), and PC (O-20:0/20:4), respectively (Figure 2C–F). These phospholipids have the potential to contain AA moiety as one of their fatty acid tails. PC aa C38:6 may correspond to PC (16:0/22:6) (Figure 2G), and PC ae C38:5 may correspond to PC (O-16:0/22:5) or PC (O-18:0/20:5) (Figure 2H). These phospholipids have the potential to contain omega-3 LC-PUFAs, such as C20:5n3 (eicosapentaenoic acid; EPA), C22:5n3 (docosapentaenoic acid; DPA), or C22:6n3 (docosahexaenoic acid; DHA) as one of their fatty acid tails. In this study, we investigated LPC(20:4) (lysophosphatidylcholine a C20:4n6) as a candidate for the SLC22A23 transporter, because LPC(20:4) was consistently and most profoundly affected by the *Slc22a23* knockout (Figure 2A).

LPC(20:4), which refers to LPC(C20:4n6) in this study, is a phospholipid that contains a single ester-linked chain of AA attached to GPC. LPC(20:4) is a water-soluble phospholipid and is known as an efficient form of phospholipid for transporting esterified AA to the brain [30]. The AA (C20:4n6) and DHA (C22:6n3) are well-known as abundant LC-PUFA in the mammalian brain. It has been reported that AA and DHA make up approximately 20% of the fatty acids in the rat brain [22,31]. AA, or its precursor linoleic acid (C18:2n6), must be obtained from dietary sources [22]. The majority of AA synthesis from linoleic acid takes place in the liver [23], after which AA must be transported to the brain. Although numerous metabolomics analyses have been conducted on SLC22 transporters, especially in knockouts of well-characterized organic anion transporters such as *Slc22a6* (*Oat1*) and *Slc22a8* (*Oat3*) [32,33,34,35,36,37,38], significant alterations in LPC(20:4) or other phospholipids containing LC-PUFA as one of their fatty acid tail(s) have not been reported yet.

Although plasma LPC(20:4) levels increased after tail-vein administration in WT rats, the levels in *Slc22a23* knockout rats did not increase and were constantly and significantly lower (Figure 4A), which was unexpected. At least three possible molecular mechanisms could explain the lack of increase and the constantly low plasma LPC(20:4) levels after intravenous administration: (1) increased uptake of LPC(20:4) by hepatocytes and enhanced biliary secretion, (2) increased lysophospholipase activity in plasma, and (3) increased renal excretion of LPC(20:4). Regarding (1) increased uptake of LPC(20:4) by hepatocytes and enhanced biliary secretion, it is widely recognized that the liver plays a major role in the removal of plasma LPC [39], and biliary secretion of LPC has been demonstrated in rat liver [40]. However, if the lower plasma level of LPC(20:4) is due to more efficient uptake into hepatocytes, why would LPC(20:4) be taken up more efficiently by hepatocytes in *Slc22a23* knockout rats? A specific transporter is required for the cellular uptake of LPC, such as MFSD2A (SLC59A1) for LPC(22:6) (lysophosphatidylcholine a C22:6n3) [15]. Although it is difficult to explain how the absence of a transporter (*Slc22a23*^−/−^) would lead to increased uptake of LPC(20:4) into hepatocytes, hepatic AA levels in *Slc22a23* knockout rats were significantly higher than those in WT rats 2 h after intravenous administration (Figure 4E). This result supports the idea of increased hepatic uptake of LPC(20:4) and subsequent AA production in *Slc22a23* knockout rats. Regarding (2) increased lysophospholipase activity in plasma, LPC(20:4) can be degraded in the circulation by lysophospholipases, including lysophospholipase A1 and lysophospholipase D [41]. However, if the lower plasma level of LPC(20:4) is due to more efficient degradation in plasma, why would lysophospholipase activity be enhanced in the absence of a transporter (*Slc22a23*^−/−^)? It is difficult to explain how the loss of a transporter (*Slc22a23*^−/−^) would result in increased lysophospholipase activity in plasma. Furthermore, plasma AA levels in *Slc22a23* knockout rats were significantly lower than those in WT rats 0.5 h after intravenous administration (Figure 4D), suggesting that efficient degradation of LPC(20:4) in plasma is unlikely. Lastly, regarding (3) increased renal excretion of LPC(20:4), the kidney, alongside the liver, is one of the most important organs for the excretion of drugs and metabolites: the liver excretes compounds into bile, whereas the kidney excretes compounds into urine [42]. Renal excretion of drugs and metabolites involves three distinct processes: glomerular filtration, active tubular secretion, and passive tubular reabsorption [42]. SLC22A23 belongs to the SLC22 family of membrane transporters, which facilitate substrate movement across membranes through passive transport or secondary active transport driven by concentration gradients [43]. Therefore, SLC22A23 could be involved in the passive tubular reabsorption of LPC(20:4), although there are currently no supporting data for this possibility. If the SLC22A23 functions in the passive tubular reabsorption of LPC(20:4) in the distal renal tubule, the reduced plasma levels observed in *Slc22a23* knockout could be simply explained by decreased reabsorption and increased urinary loss. The *Slc22a23* gene is expressed in the mammalian/rodent renal system, as indicated by the gene expression databases (https://www.bgee.org/gene/ENSMUSG00000038267 (accessed on 17 July 2026)). *Slc22a17*, which shares the highest homology with *Slc22a23*, is expressed in the distal nephron [11] and is proposed to function as a receptor that reabsorbs proteins (ex. metallothionein) via receptor-mediated endocytosis [11]. Similar to SLC22A17, SLC22A23 may mediate the reabsorption of LPC(20:4) in the distal renal tubule. Therefore, possible mechanisms could include (1) increased uptake of LPC(20:4) by hepatocytes and enhanced biliary secretion, and/or (3) increased renal excretion of LPC(20:4). Further studies are warranted to elucidate the precise molecular mechanism. Consequently, we have observed a reduction in LPC(20:4) levels in the plasma of *Slc22a23* knockout rats (AA). Additionally, there was a reduction in LPC(18:2) in the plasma of these knockouts (B), suggesting that the uptake of linoleic acid (C18:2n6), a precursor of AA synthesis, is also impaired in *Slc22a23* knockout rats. This further supports the observed reduction in LPC(20:4) in the plasma of *Slc22a23* knockout rats.

Using ^14^C radioactivity, researchers examined the brain uptake of LPC(20:4) (2-acyl-1-lysoPC a C20:4n6) and unesterified AA in rats [30]. The brain uptake of the LPC(20:4) reached a plateau within 30 min and was 6 to 10 times higher than that of unesterified AA [30]. Transport of water-soluble compounds across the plasma membrane, particularly across the blood–brain barrier, requires specific transporters expressed on the plasma membrane. In the case of DHA, MFSD2A (SLC59A1) is proposed to facilitate the transport of LPC(22:6) (lysophosphatidylcholine a C22:6n3) across the blood–brain barrier, allowing DHA to be delivered to the brain [15]. In their study, the amount of DHA in the brain was reduced in *Mfsd2a* knockout mice, but the level of AA was not affected. This suggests that LPC(20:4) is transported across the blood–brain barrier by a transporter other than MFSD2A (SLC59A1). Our mass spectrometry analysis in this study suggests that the SLC22A23 transporter may function as a transporter of LPC(20:4), potentially playing a role in its transport to the brain. Additionally, because the SLC22A23 protein is highly conserved between humans and rats (with an alignment score of 91.3 calculated using Clustal W), the findings of this study are likely relevant to human biology.

### 4.2. Administration of LPC(20:4) Induces Memory Enhancement in Slc22a23-Proficient Rats

Following the observation of a substantial decrease in arachidonic acid (AA) in aged rats [44], numerous studies have shown that feeding an AA-containing diet can restore brain functions in aged rats (typically 21–24 months old rats). These improvements include restoration of long-term potentiation [45,46], hippocampal neuron membrane fluidity [47], and spatial cognition [48,49]. Furthermore, dietary supplementation of AA, along with DHA, has been shown to improve cognitive dysfunction in elderly humans with amnesia [50]. In a human cohort, Snowden et al. conducted plasma lipidomics at age 58 and brain MRI scanning 10 years later (at age 68). Lysophosphatidylcholines, including LPC(20:4) and LPC(22:6), were identified as predictors of brain ageing [51]. Maekawa et al. demonstrated that feeding a diet supplemented with AA (but not DHA) for 4 weeks after birth promotes neurogenesis in young wild-type rats [52]. In our previous studies [53,54], we investigated the effects of esterified diacyl (aa) phospholipids, etherified (at the sn-1 position) and esterified (at the sn-2 position) diacyl (ae) phospholipids, including alkyl(O)-acyl, alkenyl(P)-acyl (plasmalogen), on animal behavior. We identified both solitary and synergistic effects of different hydrophilic and hydrophobic phospholipid moieties on rat behaviors. While AA was utilized at the sn-2 position in those studies, the effects of a single AA moiety within a phospholipid (such as LPC(20:4)) on animal behavior had not been examined. In contrast, Sugasini et al. reported that dietary DHA administered as LPC(22:6), rather than as non-esterified DHA, enriches brain DHA levels and enhances spatial memory acquisition in the MWM test [14]. Therefore, we investigated the effects of a single AA moiety within a phospholipid (such as LPC(20:4)) on animal behavior in the present study.

2-Arachidonoylglycerol (2-AG) is a neurotransmitter that acts as a full agonist of the G-protein coupled receptors CB1 and CB2 [55,56]. 2-AG is classified as an endogenous cannabinoid, distinguishing it from Δ-9-tetrahydocannabinol (THC), the main psychoactive compound in cannabis sativa, which is known for its psychotropic effects [57]. 2-AG functions as a retrograde inhibitor of neurotransmitter release from presynaptic neurons by interacting with CB1 receptors, which are present in both excitatory and inhibitory presynaptic terminals [58,59]. The interaction between 2-AG and CB1 receptors underpins 2-AG’s role in memory, pain, anxiety, mood, stress regulation, hyperexcitability control, neuroprotection, and addiction. Recently, Briand-Mésange et al. [60] proposed an extracellular pathway for synthesizing 2-AG, involving the direct conversion of 2-acyl-1-lysoPC a C20:4n6 by ecto-nucleotide pyrophosphatase/phosphodiesterase 6 (ENPP 6) or ENPP7, which are members of the phospholipase C family. Morita et al. [61] have provided strong evidence that GPCs are the natural substrates of ENPP6, enabling oligodendrocytes to acquire the choline necessary for myelin biosynthesis. Therefore, ENPP6 may facilitate cleavage between glycerol and phosphate in 2-acyl-1-lysoPC a C20:4n6, leading to the production of 2-AG.

In our previous study [4], we found that *Slc22a23* knockout rats exhibit a lean phenotype, increased spontaneous exploratory movements in the OF, and reduced hippocampal volume, suggesting that *Slc22a23* is involved in metabolism and neurodevelopment. In this study, we conducted animal behavioral tests after administration of LPC(20:4), a candidate substrate of the SLC22A23 transporter. Intravenous LPC(20:4) administration showed further reduction in total distance travelled during the OF test in *Slc22a23*^+/+^ rats at 12 weeks of age (Figure 6D). In the NOR test, access to the novel object was significantly decreased during the latter half in *Slc22a23*^+/+^ at 8 weeks of age and *Slc22a23*^−/−^ at 12 weeks of age (Figure 7D). After the rats explored the objects during the first 5 min (first half), their access to the novel object was significantly reduced during the subsequent 5 min (latter half) in the LPC (20:4) administered groups. We therefore interpret these results as indicative of memory enhancement. In the SI test, sociability to a familiar rat was significantly reduced 2 and 6 h after injection in *Slc22a23*^+/+^ rats at 9 weeks, and also 6 h after injection in *Slc22a23*^−/−^ rats at 9 weeks (Figure 8B). In the LPC (20:4) administered groups, 9-week-old rats showed significantly reduced interaction with rat A on day 3 compared with day 2. We interpret these results as indicative of enhanced social memory. However, this effect was not observed in 13-week-old rats, likely due to age-related differences. In the MWM test, LPC(20:4) administration during the reversal phase significantly shortened the latency to the target in *Slc22a23*^+/+^ rats (Figure 10A,B,D) and also notably in *Slc22a23*^−/−^ rats (Figure 10D). LPC(20:4) administration can be effective in both young (9 weeks) and fully mature (13 weeks) adult rats in the MWM test. LPC(20:4) was administered 6 h before the trials on days 1 and 2 of the reversal phase. The LPC(20:4) administration shortened the latency to reach the platform (Figure 10) and swim distance from start to goal (Figure 11). These improved performances were observed specifically in the first trial on day 3 (Figure 10D and Figure 11D). We therefore assume that memory consolidation is the main effect of LPC(20:4) administration. We also observed faster swim speed from start to goal in WT rats on day 3 (Figure 12B). The difference mainly came from trials 2–4 (Figure 12F), suggesting that LPC(20:4) administration also improves learning and recall across trials. Overall, these results suggest that LPC(20:4) administration enhances memory acquisition. LPC(20:4) administration in *Slc22a23*^+/+^ rats appeared more effective, although it also showed significant effects in *Slc22a23*^−/−^ rats. Further studies are warranted to clarify the underlying mechanisms. Its benefits may result from supplying AA, 2-AG, choline or a combination of these compounds to the brain.

In the MWM test, we observed significant differences mainly on day 3, one day after the last injection, during the reversal phase (Figure 10B,D, Figure 11D and Figure 12B,F). From day 4, the rats reached the platform very quickly, likely due to a ceiling effect, indicating that the task was too easy for them. In future experiments, a larger pool or a smaller platform should be used to increase task difficulty. Notably, in the acquisition phase of the MWM test at 8 weeks of age, randomly separated naïve rats exhibited significant differences. For example, trial 1 of the acquisition phase in *Slc22a23*^+/+^ rats differed significantly between naïve (C) and naïve (L) groups (Figure S1C). Significant differences between naive rat groups were also observed in swim speed on day 1 (Figure S3A,E). These results suggest the potential for false positives in the MWM analysis. Regarding the carryover effect of LPC(20:4) administration at 9 weeks of age, no significant differences were observed on day 1 of the acquisition phase at 12 weeks of age (Figures S1B, S2B and S3B), suggesting that the carryover effect from LPC(20:4) administration at 9 weeks of age is likely to be minor. However, *Slc22a23*^−/−^ rats in the acquisition phase at 12 weeks of age showed shorter latencies to the target in trials 2–4 on day 3 (Figure S1F) and faster swim speeds on days 2 and 5 (Figure S3B,D,F). Thus, carryover effects potentially may exist, particularly in *Slc22a23*^−/−^ rats. Furthermore, this study was not conducted in a blinded manner and may therefore contain potential sources of bias.

### 4.3. Elucidating the Mechanism of Action of SLC22A23 Is Crucial and Urgent

It was unclear whether SLC22A23 directly facilitates the incorporation of LPC(20:4) into cells, or if SLC22A23 influence the in vivo kinetics of LPC(20:4) indirectly. To address this question, we conducted an LPC(20:4) incorporation assay using primary hippocampal neurons. At a physiological LPC(20:4) concentration (10 µM), no significant difference in incorporation was observed between SLC22A23-expressing WT cells and *Slc22a23* knockout cells (Figure 13A). However, when a higher concentration (100 µM) of LPC(20:4) was applied, incorporation was significantly higher in WT cells than in *Slc22a23* knockout cells 30 min after addition (Figure 13B), suggesting that the SLC22A23 transporter can facilitate LPC(20:4) uptake into the cells. These results also suggest that additional factors, such as fatty acid binding proteins, may facilitate the interaction between LPC(20:4) and SLC22A23 in vivo. At present, SLC22A23 remains an orphan membrane transporter. The gene with the highest homology to *Slc22a23* is *Slc22a17*. Based on the sequence analysis, both *Slc22a23* and *Slc22a17* are classified as non-common types of *Slc22* transporters and belong to the organic anion transporter-related subclade (OAT-related subclade) [5]. SLC22A17 is known to interact with Lipocalin-2 (a binding protein for hydrophobic small molecules) through its N-terminal extracellular domain, facilitating the internalization of Lipocalin-2 into the cell via receptor-mediated endocytosis [10,11,62]. Since *Slc22a17 (Ngalr)* knockout mice are reported to be embryonic lethal (as noted in Schröder et al.’s review stating that “Homozygote *Ngalr* mutant mice are embryonic lethal (Yukio Nakamura, personal communication)”) [63], our study employing *Slc22a23* knockout rats is expected to elucidate the in vivo function of the OAT-related subclade of the *Slc22* family transporters. Additionally, several extracellular lipid-binding proteins have been confirmed in plasma, including Lipocalin-2 [64], FABP1 [65,66], FABP3 [67,68,69], FABP4 [70,71,72], and RBP4 [73]. SLC22A23 may interact with one of these extracellular lipid-binding proteins through its N-terminal extracellular domain and function as a transporter facilitating cellular uptake. Since *Slc22a23* and *Slc22a17* share the highest homology, they may have redundant functions in vivo. To elucidate the role of SLC22A23 in the pharmacokinetics of LPC(20:4) in vivo, both *Slc22a23* and *Slc22a17* should be examined simultaneously. Because *Slc22a17* knockout rats are likely embryonic lethal, as observed in mice, generating conditional *Slc22a17* knockout rats will be warranted for future research.

## Data Availability

The original contributions presented in this study are included in the article/Supplementary Material. Further inquiries can be directed to the corresponding author.

## Supplementary Materials

The following supporting information can be downloaded at: https://www.mdpi.com/article/10.3390/pharmaceutics18080975/s1, Table S1: Result of rat plasma mass spectrometry under organic extraction; Table S2: Result of rat plasma mass spectrometry under water extraction; Table S3: The descriptive statistics of the data shown in Figure 4, Figure 5, Figure 6, Figure 7, Figure 8, Figure 9, Figure 10, Figure 11, Figure 12 and Figure 13; Figure S1: Latency to the target in the acquisition phase of the MWM, Figure S2: Swim distance from start to goal in the acquisition phase of the MWM, and Figure S3: Swim speed from start to goal in the acquisition phase of the MWM.

## Abbreviations

The following abbreviations are used in this manuscript:

*Slc22a23*	solute carrier family 22 member 23
LPC(20:4)	lysophosphatidylcholine C20:4n6
LPC(18:2)	lysophosphatidylcholine C18:2n6
LPC(22:6)	lysophosphatidylcholine C22:6n3
GPC	glycerophosphocholine
AA	arachidonic acid (C20:4n6)
DHA	docosahexaenoic acid (C22:6n3)
OF	open field
NOR	novel object recognition
SI	social interaction
MWM	Morris water maze
