# Supplementary material for: Slc22a23 Proficiency Influences Rat Behavioral Responses After Lysophosphatidylcholine C20:4n6 Administration
Source: Pharmaceutics. 2026 Aug 8;18(8):975. doi: 10.3390/pharmaceutics18080975 (PMC13516374; doi:10.3390/pharmaceutics18080975)
Supplement: Supplementary file 1 [file pharmaceutics-18-00975-s001.zip › Supplementary files/Figure S1, S2 and S3.pdf]

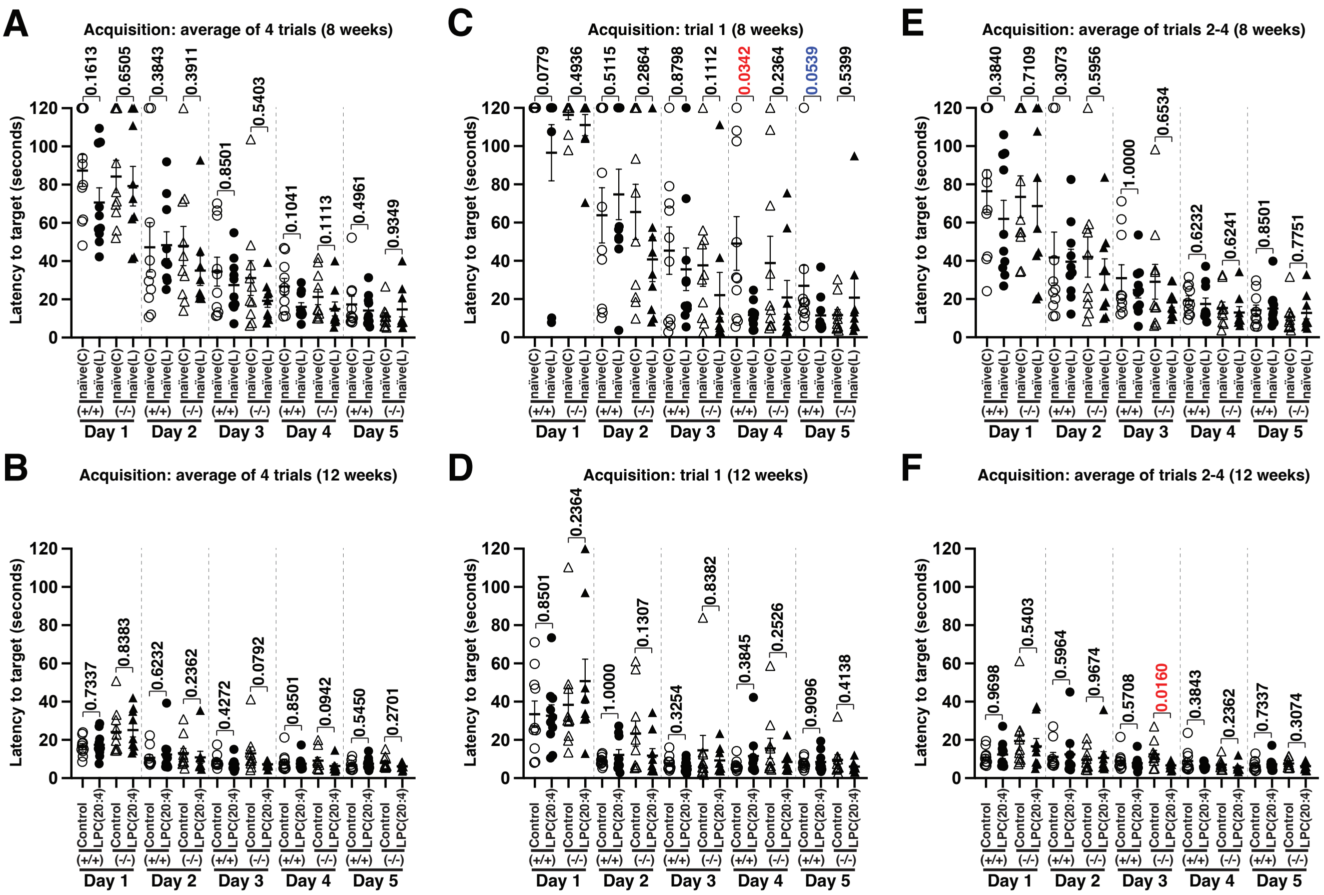

Figure S1. Latency to the target in the acquisition phase of the MWM: Data from the acquisition phase of the MWM were analyzed and plotted using the same procedure described in the legend of Figure 10.

Figure S1

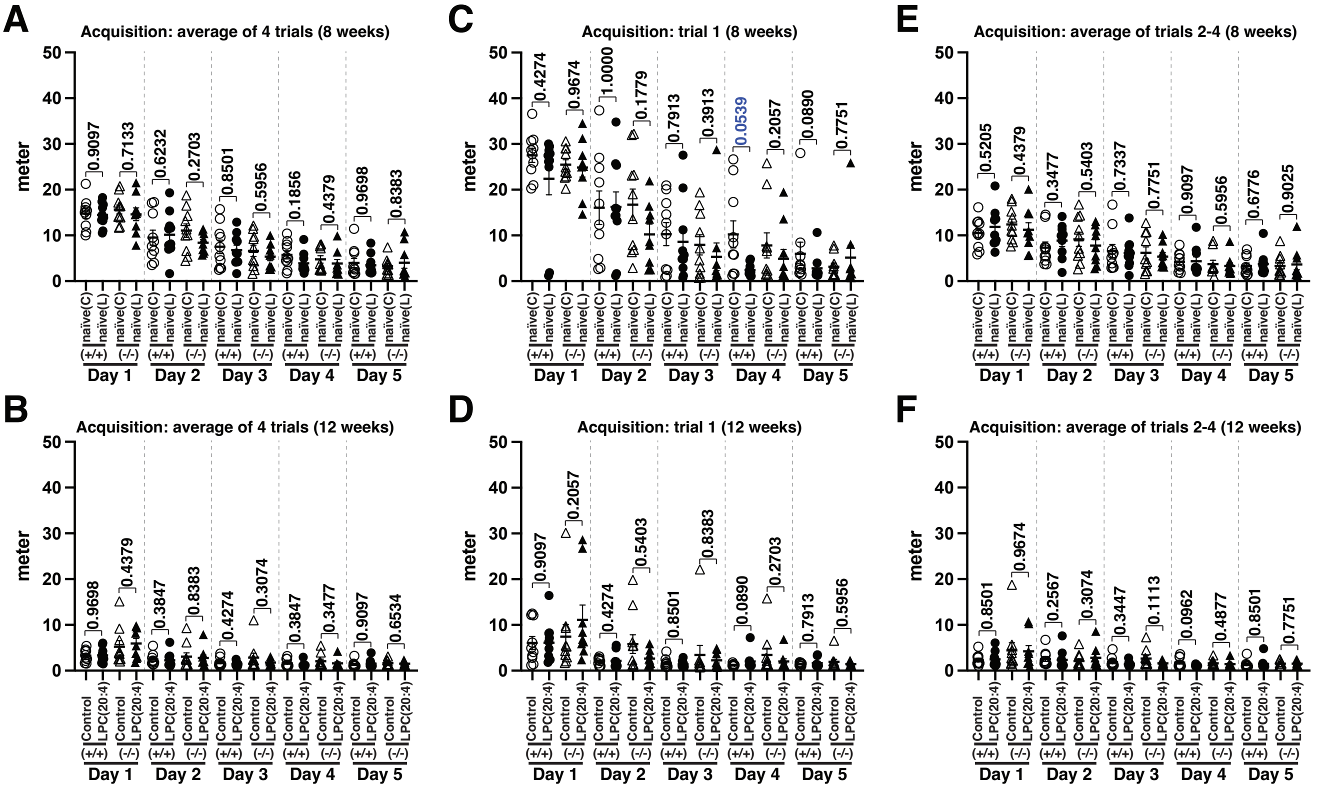

Figure S2. Swim distance from start to goal in the acquisition phase of the MWM: Data from the acquisition phase of the MWM were analyzed and plotted using the same procedure described in the legend of Figure 11.

Figure S2

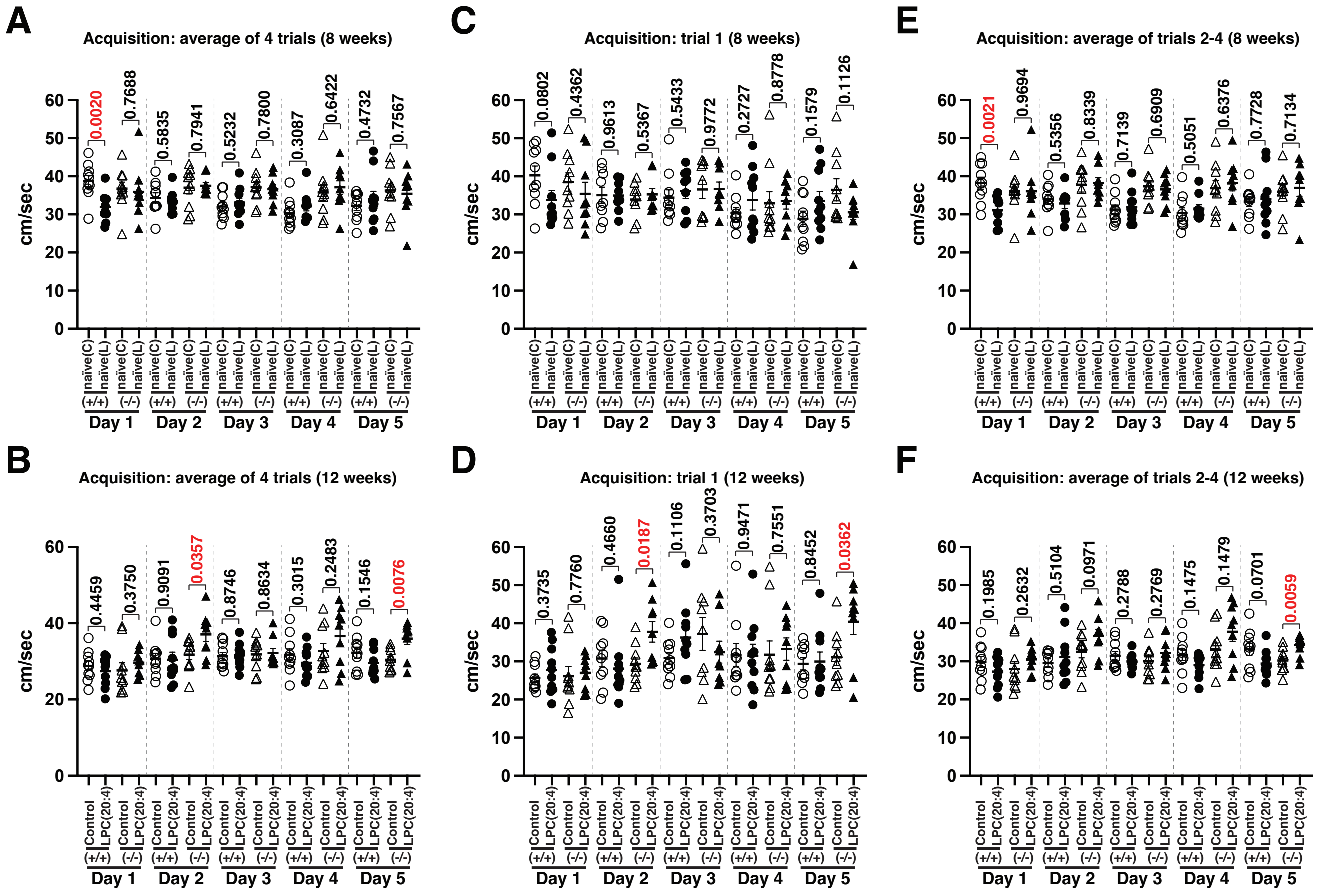

Figure S3. Swim speed from start to goal in the acquisition phase of the MWM: Data from the acquisition phase of the MWM were analyzed and plotted using the same procedure described in the legend of Figure 12.

Figure S3
